# Is There a Relationship between Fish Cannibalism and Latitude or Species Richness?

**DOI:** 10.1371/journal.pone.0169813

**Published:** 2017-01-25

**Authors:** Larissa Strictar Pereira, Friedrich Wolfgang Keppeler, Angelo Antonio Agostinho, Kirk O. Winemiller

**Affiliations:** 1 Nupelia, Universidade Estadual de Maringá, Av.Colombo, Maringá, PR, Brazil; 2 Department of Wildlife and Fisheries Sciences, Texas A&M University, College Station, TX, United States of America; Uppsala Universitet, SWEDEN

## Abstract

Cannibalism has been commonly observed in fish from northern and alpine regions and less frequently reported for subtropical and tropical fish in more diverse communities. Assuming all else being equal, cannibalism should be more common in communities with lower species richness because the probability of encountering conspecific versus heterospecific prey would be higher. A global dataset was compiled to determine if cannibalism occurrence is associated with species richness and latitude. Cannibalism occurrence, local species richness and latitude were recorded for 4,100 populations of 2,314 teleost fish species. Relationships between cannibalism, species richness and latitude were evaluated using generalized linear mixed models. Species richness was an important predictor of cannibalism, with occurrences more frequently reported for assemblages containing fewer species. Cannibalism was positively related with latitude for both marine and freshwater ecosystems in the Northern Hemisphere, but not in the Southern Hemisphere. The regression slope for the relationship was steeper for freshwater than marine fishes. In general, cannibalism is more frequent in communities with lower species richness, and the relationship between cannibalism and latitude is stronger in the Northern Hemisphere. In the Southern Hemisphere, weaker latitudinal gradients of fish species richness may account for the weak relationship between cannibalism and latitude. Cannibalism may be more common in freshwater than marine systems because freshwater habitats tend to be smaller and more closed to dispersal. Cannibalism should have greatest potential to influence fish population dynamics in freshwater systems at high northern latitudes.

## Introduction

Ever since Ricker [1] concluded that cannibalism is the ultimate mechanism of density dependence, ecologists have debated the influence of cannibalism on population regulation [2–5]. Fitness advantages of cannibalism have been demonstrated for taxa ranging from arthropods to tetrapod vertebrates [6,7]. In fact, cannibals obtain high-quality nutrition while eliminating potential competitors and predators [8], and they tend to grow faster than non-cannibals [9,10]. However, there appears to be little consensus about whether or not cannibalism is common and its ecological and evolutionary importance.

Cannibalism has been reported for many teleost fishes [11], but most accounts involve captive fish with limited food supply or options [12–14]. Based on currently available information for wild fishes, cannibalism seems to be most common in high latitudes [15], including Arctic char (*Salvelinus alpinus*), pike (*Esox lucius*) and perch (*Perca fluviatilis*). In fact, cannibalism was shown to be more common in Arctic than temperate populations of Arctic char [16]. Cannibalism reports often come from lakes with few fish species, including some containing only a single species [15,17–20]. More cannibalism was recorded for Arctic char inhabiting a lake that had no other fish species when compared to char from two other lakes containing several other fish species [12]. In an experiment with ponds containing only perch and roach (*Rutilus rutilus*), juvenile perch cannibalized smaller conspecifics in years when roach hatched earlier than perch and were too large to be suitable as prey for the latter [21]. On the other hand, in species-rich communities at low latitudes, cannibalism is rarely reported, and when reported, seems to be limited to few species (e.g., *Cichla kelberi* and *Plagioscion squamosissimus*) [22,23].

**Fig 1:**
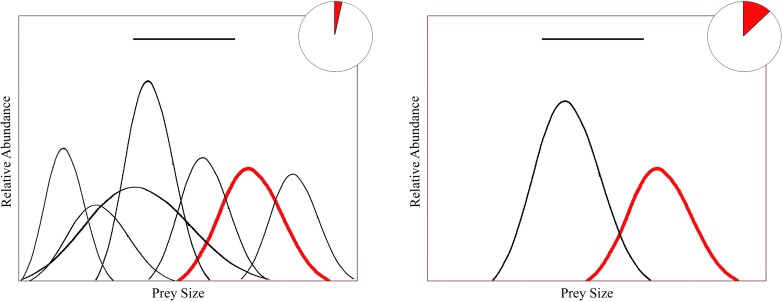
Predicted influence of prey species diversity on probability of prey encounter rates and cannibalism. In a species-rich assemblage (A), low relative abundance of conspecific prey (red curves) results in low rates of encounter with conspecifics relative to heterospecific prey (black curves) and a low rate of cannibalism. In a species-poor assemblage (B), conspecific prey comprise a greater percentage of available prey, and encounter rates with conspecifics and cannibalism would be higher. Curves represent size frequency distributions of various prey. Heavy black horizontal line represents the range of prey sizes consumed by the predator. Pie charts represent dietary proportions of conspecific (red area) and heterospecific prey (white area).

Why should cannibalism be more common in high latitude systems with few species? If cannibalism is a function of the probability of encountering conspecifics relative to encounters with other species that also are potential prey, then, assuming all else being equal, frequency of cannibalism should be inversely related with species richness. For predators in species-poor assemblages, the spectrum of available food resources would include a significant percentage of conspecific prey (Fig 1). With increasing species richness, the diversity of available prey would increase and the percentage (relative abundance) and probability of encounters with conspecific prey would decline [24–26]. Given that species richness and diversity of freshwater and marine fishes declines with latitude [27–29], incidence of cannibalism should increase with latitude. These predictions were tested by analyzing a global database of fish cannibalism compiled from a literature survey of fish diets. Freshwater and marine fishes were tested separately and results compared, as were fishes from Northern and Southern hemispheres.

## Methods

### Literature search and data extraction

Using the ISI *Web of Science*, a literature survey was conducted using the keywords “fish” and “cannibalism” and November 2015 as the final date for inclusion. From the results of this search, studies performed in captive environments (e.g. laboratory, cages, experimental ponds) were excluded, whereas studies of natural environments (anthropogenically impacted or not) were retained. A second search was performed using the key words “trophic ecology of fish” filtered by the keyword “feeding”. From the results of these searches, we eliminated duplicate accounts, reports from captive fish, and reports that did not contain original observations or direct estimates of diet (e.g., isotopic analysis, modeling, or reviews). From the remaining studies, the following information was recorded: (*i*) presence or absence of cannibalism, (*ii*) realm (freshwater or marine), (*iii*) name of the target species, (*iv*) number of reported cannibalism cases, and (*v*) geographical coordinates of the study.

Fish were placed into general trophic groups based on descriptions provided by publications obtained from the search, and complemented by information in *FishBase* [30]. Because the dataset included publications dating to 1939, *FishBase* also was used to update taxonomy and eliminate synonyms. When not reported in the publications, geographical coordinates were obtained from *Google Earth* (http://www.google.fr/intl/fr/earth/index.html) based on location descriptions. At each location, the number of fish species in the lake or drainage subbasin was obtained from *Fish-SPRICH* [31,32]; these estimates only could be made for freshwater locations.

### Data analysis

Relationships between cannibalism occurrences with fish species richness and latitude were evaluated through generalized linear mixed models (GLMM), using the binomial error distribution, logit link function, and Gauss-Hermite quadrature procedure. It should be noted that it is not assumed, a priori, that latitude has a cause-effect relationship with occurrence of cannibalism, but rather that latitude represents a spatial dimension that is associated with other variables (e.g., productivity, species richness) that can affect frequency of cannibalism. Cannibalism occurrence was recorded as the presence or absence of cannibalism by a given species at a given location based on a given literature report. Model sets were built separately for Northern and Southern hemispheres and for freshwater and marine realm, resulting in four different combinations: Northern/freshwater, Southern/freshwater, Northern/marine and Southern/marine. Because marine species richness data were not available, the species richness factor was only included in the analysis of the freshwater dataset. The variable *genus* was used as a random factor in order to deal with the lack of independence among sampling units caused by phylogenetic relatedness (e.g., species of the same genus tend to have similar behavior and diet) [33,34]. Although conclusions were similar, genus instead of species identity was adopted (the dataset includes multiple observations for some species) as random variable to improve the accuracy of the parameters, residual distribution, and goodness of fit. Previous comparisons using the Conditional Akaike Information Criterion (cAIC) [35,36] found that inclusion of *genus* as a random variable in models improved the goodness of fit (S1 Table). Richness data were log transformed to reduce undue influence of extreme values.

A merged dataset was created for analysis to compare the slopes of the cannibalism–latitude regression of fishes from freshwater versus marine realms in the Northern Hemisphere (latitude was not an important predictor in the Southern Hemisphere as reported below). For this merged dataset, three fixed factors were analyzed: latitude, realm (freshwater or marine) and their interaction. Genus was also included as a random variable. All carnivorous species described in reviewed publications (n = 2,314), whether they were cannibals or not, were included to generate models. Diagnostic residual plots (S1 Fig) and overdispersion tests [37] were performed along with the GLMM analyses to confirm model assumptions. Potential collinearity between latitude and richness (freshwater dataset) was evaluated using the variance inflation factor (VIF) [38].

For each hemisphere and realm, multiple models that tested all possible combination of fixed variables, including null models without fixed variables, were created. The random variable *genus* was maintained in all models. Then the best model was selected using the Akaike information criterion based on marginal likelihood (AIC). AIC was chosen rather than traditional likelihood ratio tests (LWR) because the latter is usually too conservative (high chance of type II error) and less flexible for multiple model comparisons [39,40]. Only models with delta AIC < 2 were selected for further descriptive statistics (R^2^ and CI of parameters). In order to quantify the goodness-of-fit of each model, the marginal R^2^ (R^2^_m_; variance explained by fixed factors) and conditional R^2^ (R^2^_c_; variance explained by both fixed and random factors) were derived according to the method of Nakagawa and Schielzeth [41]. Confidence intervals for the parameters of each model were calculated using the percentile method [42]. Generalized linear mixed models were computed in the R package “lme4” [43], overdispersion tests were performed using the R package “blmeco” [37], and confidence intervals for the model parameters were generated in the R package “boot” [44].

## Results

**Fig 2:**
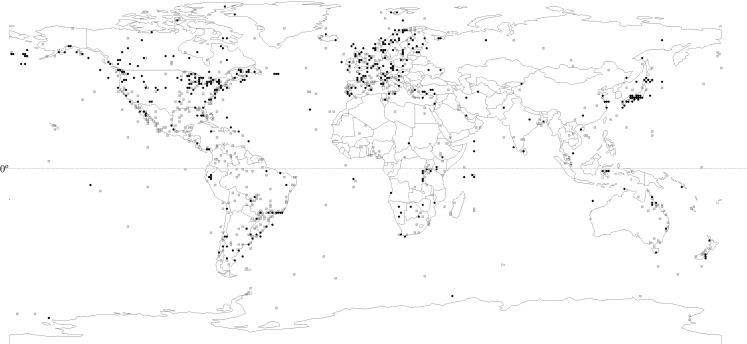
The global distribution of cannibalism occurrence based on a survey of 1,270 literature sources. Points represent the geographical coordinates of each study included in the literature review. Black dots represent studies reporting cannibalism and open dots denote absence of reported cannibalism. To avoid overlapping points, 234 coordinates were not plotted, including 134 for absence and 100 for presence of cannibalism.

The literature survey produced 1,270 publications reporting fish dietary studies. Among 4,100 fish populations encompassing 2,314 species for which there were dietary reports, 10% (237 species) revealed 618 cases of cannibalism (Fig 2—data appear in S2 Table).

**Fig 3:**
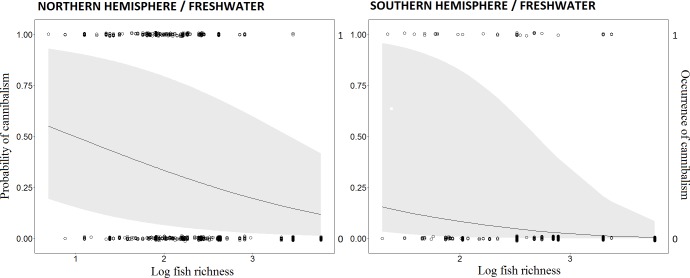
Generalized linear mixed models (GLMM) for the relationship between cannibalism occurrences and freshwater fish richness. Northern hemisphere (left side) and southern hemisphere (right side). Lines are the predicted cannibalism probability along richness gradients according to each model. Shaded areas around probabilities lines represent the 95% CI. Dots are observed data for absence (0) or presence (1) of cannibalism according to fish species richness.

Fish richness and latitude were weakly correlated in the dataset for freshwater fishes of the Southern Hemisphere (Spearman’s rank correlation = -0.23) and were moderately correlated (Spearman’s rank correlation = -0.56) in the Northern Hemisphere, which did not reveal concern for collinearity in any model (VIF < 1.3). Cannibalism occurrence, recorded as at least one report of cannibalism at a given study location, was inversely associated with species richness in freshwater systems (Fig 3). Species richness was a fixed variable in the best-fit models for both Northern and Southern hemispheres (Table 1), and had coefficient intervals not encompassing zero, suggesting consistent relationships (Table 2).

**Table 1:** Best models (GLMM) for the occurrence of cannibalism for both freshwater and marine realm in Northern and Southern hemispheres.

Realm	Hemisphere	Model	AIC	Delta AIC	AIC weights
Freshwater	North	1 + Latitude + Richness	755.47	0	0.71
		1 + Latitude	757.24	1.76	0.29
		1 + Richness	785.46	29.99	0
		1	804.63	49.15	0
	South	1 + Richness	226.78	0	0.72
		1 + Latitude + Richness	228.74	1.96	0.27
		1	236.69	9.91	0.01
		1 + Latitude	238.31	11.52	0
Marine	North	1 + Latitude	963.74	0	1
		1	986.72	22.98	0
	South	1 + Latitude	267.43	0	0.56
		1	267.92	0.48	0.44

The constant 1 included in all models is the intercept. Because marine species richness data were lacking, the species richness factor was only included in models derived from the freshwater dataset.

**Table 2:** Parameter estimation of the best models (GLMM with delta AIC lower than 2) predicting occurrence of cannibalism for both freshwater and marine realm in Northern and Southern hemispheres.

Realm	Hemisphere	Model Rank	Parameter	Estimate	95% CI	R^2^—Marginal and Conditional
Freshwater	North	1°	Intercept	-2.78	-3.72 to -2.007	0.29–0.47
			Latitude	0.05	0.04 to 0.065	
			Richness	-0.04	-0.09 to -0.005	
		2°	Intercept	-3.55	-4.14 to -2.99	0.25–0.45
			Latitude	0.06	0.045 to 0.07	
	South	1°	Intercept	-4.02	-8.76 to -2.50	0.10–0.79
			Richness	-0.13	-0.20 to -0.07	
		2°	Intercept	-4.14	-9.42 to -2.34	0.10–0.79
			Latitude	0.005	-0.05 to 0.04	
			Richness	-0.12	-0.22 to -0.075	
Marine	North	1°	Intercept	-4.94	-6.23 to -3.96	0.06–0.59
			Latitude	0.05	0.03 to 0.07	
	South	1°	Intercept	-2.74	-4.90 to -1.40	0.02–0.62
			Latitude	-0.025	-0.07 to 0.01	
		2°	Intercept	-3.475	-6.125 to -2.60	0–0.68

Confidence intervals (CI) based on the percentile method are given for each parameter. Marginal coefficient of determination (R^2^) represents the variance explained by fixed factors, while conditional R^2^ represents the variance explained by both random (species) and fixed factors.

**Fig 4:**
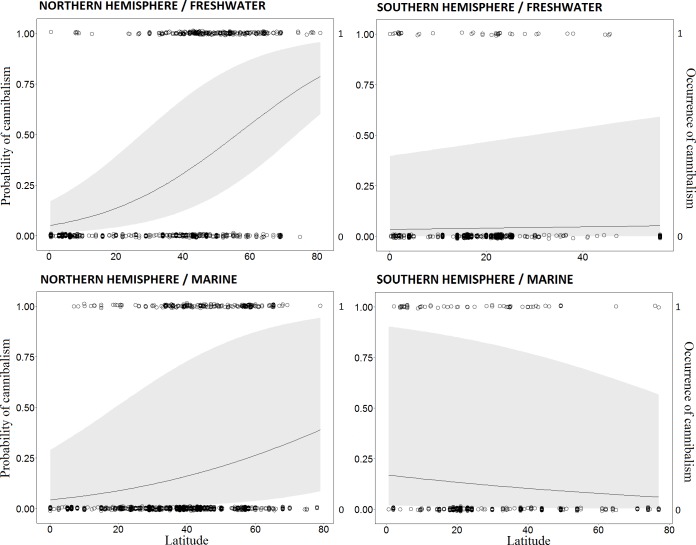
Generalized linear mixed models (GLMM) for the relationship between cannibalism occurrence and latitude. Freshwater (top figures) and marine (bottom figures) realm in the Northern (left figures) and Southern hemisphere (right figures). Lines are the predicted cannibalism probability along latitudinal gradients according to each model. Shaded areas around probabilities lines represent the 95% CI. Dots are observed data for absence (0) or presence (1) of cannibalism according to fish species richness.

Cannibalism was positively correlated with latitude in both freshwater and marine realms within the Northern Hemisphere (Fig 4, Tables 1 and 2). The model that included only latitude as the fixed variable had a lower ΔAIC than the model that included only species richness for Northern Hemisphere freshwater fish (1.76 vs. 29.99, respectively), indicating that latitude is a more reliable predictor of cannibalism (Table 1). The interaction between latitude and realm (freshwater vs. marine) was more important than either fixed variable alone in the best-fit model comparing slopes of the cannibalism–latitude relationships for freshwater and marine realms in the Northern Hemisphere (AIC weights = 0.61; Table 3). The freshwater slope was steeper than the marine slope (Latitude: Marine realm = -0.02; Fig 4). The relationship between cannibalism and latitude was weak and inconsistent for freshwater and marine fishes in the Southern Hemisphere (Fig 4; Tables 1 and 2). Marginal R^2^ values were low (<0.29; Table 2) for all models evaluated, and conditional R^2^ values were medium to high (>0.79; Table 2).

**Table 3:** Best models (GLMM) for the occurrence of cannibalism in the Northern Hemisphere.

Model	AIC	ΔAIC	AIC weights
1 + Latitude + Realm + Latitude * Realm	1748.16	0	0.61
1+ Latitude + Realm	1749.02	0.86	0.39
1 + Latitude	1768.40	20.23	0
1 + Realm	1822.13	73.97	0
1	1829.06	80.89	0

The constant 1 is included in all models as the intercept. The coefficient intervals and R^2^ for the latitude variable in each realm (marine and freshwater) can be found in Table 2.

## Discussion

The occurrence of cannibalism was lower in freshwater fish assemblages with higher species richness. This relationship was stronger for the dataset from the Northern Hemisphere (range of probability of cannibalism occurrence, as predicted by the model, ranging from ca. 13 to 55) than the Southern Hemisphere (range of probability of cannibalism occurrence from 0 to 16; see Fig 3). This difference between Northern and Southern hemispheres could be due to a steeper gradient of species richness among freshwater habitats in the Northern Hemisphere. Species richness of freshwater fish tends to be lower in the Northern than in the Southern Hemisphere [45], being a result of past and contemporary environmental and biotic conditions, including gradients of temperature and rainfall, environmental productivity, thermal tolerance, competition, and predation [27,46–49]. Several of the fish dietary studies from the Northern Hemisphere were conducted in areas with fewer than 10 species (lowest value: a single fish species in Lake Hazen, Canada), whereas only two studies from the Southern Hemisphere had fewer than 10 species (lowest value: 7 species in Patagonia, Argentina). Latitude was more important than richness to explain cannibalism in freshwaters of the Northern Hemisphere. This result suggests that another factor (or factors) correlated with latitude could influence differences in cannibalism frequency across large spatial scales. One of them is the higher proportion of piscivorous fishes in freshwater systems at higher latitudes in the Northern Hemisphere [50]. If piscivores are more prone to cannibalism as some have claimed [7,11], then greater proportions of piscivores at higher latitudes would promote a latitudinal gradient of cannibalism. Omnivory and herbivory appear to be more common among tropical than temperate freshwater fishes [50,51], which could contribute to lower incidence of cannibalism at lower latitudes [11]. Conversely, if most cannibalism involves predation on egg and larval stages, then omnivorous and herbivorous fishes also are capable of cannibalism and assemblage trophic structure would be less important. This hypothesis is difficult to test because eggs and larvae are difficult to identify morphologically and are digested rapidly. Future research should examine whether higher proportions of piscivores at higher latitudes increases cannibalism, and if other factors also affect the latitudinal gradient.

The relationship between cannibalism and latitude differed between hemispheres for marine fishes. In the Northern Hemisphere cannibalism revealed a weak negative relationship with latitude, whereas no relationship was evident for marine fishes in the Southern Hemisphere. This discrepancy could again be explained by a stronger species richness gradient for marine fishes in the Northern Hemisphere [46]. With more than 3,000 species described, the Australia-East Indies region has the world’s highest marine fish richness [52–54]. Within the Southern Hemisphere, this region extends from the equator to ~45°S. In contrast, the region within the Northern Hemisphere with greatest marine fish richness (ca. 700 species) is the Caribbean Sea [55], a zone that extends from the equator to only about 18°N.

In the Northern Hemisphere, the slope of the cannibalism–latitudinal relationship for freshwater fishes was steeper than the slope for marine fishes (Fig 4). The overall difference in net productivity between marine and freshwaters ecosystems might partially account for this difference. Whereas productivity in freshwater declines between tropical and temperate latitudes, marine productivity generally increases [56]. Higher marine productivity near the poles may support greater fish biomass and local species diversity [57]. Conversely, lower freshwater productivity and species richness at higher latitudes may strengthen the cannibalism gradient. Another explanation for the difference in cannibalism gradients in freshwater and marine realms is the greater size and spatial connectivity of marine ecosystems that facilitate dispersal and local diversity [58]. Marine fish eggs and larvae that drift in currents are often spatially segregated from conspecific juveniles and adults [59], reducing opportunities for cannibalism. Fish assemblages in freshwater habitats tend to be dispersal limited and insular compared to marine fishes [60]. If dispersal limitation decreases access to profitable foraging habitats during periods of low food availability, cannibalism may increase if juvenile stages are unable to segregate spatially from larger conspecifics [24].

With the exception of the best-fit models for freshwater fishes in the Northern Hemisphere, GLM models had marginal R^2^ values (R^2^_m_) proportionally lower than the corresponding conditional R^2^ values (R^2^_c_). This suggests that genus may explain more of the variability of cannibalism occurrence than latitude or species richness. As a group, teleost fishes display tremendous interspecific variation with respect to morphology, physiology, behavior, habitat use and life history [45,61–63], and some fish lineages would logically be expected to be more prone to cannibalism than others [4]. For example, cannibalism often has been observed in fish that are territorial or exhibit parental care [64]. Therefore, predictions of cannibalism occurrence in fish based only on latitude or species richness may be subjected to high levels of uncertainty caused by the inherent diversity of species traits (e.g., feeding habits, parental care behavior). Use of mixed models provided a means to control for a lineage-specific (or species-specific) influence on relationships between cannibalism with species richness and latitude. Alternatively, future studies may explore the variation of cannibalism occurrence along latitudinal gradients in specific lineages (e.g., genus with strong niche conservatism signal; 34) or in species with broad geographical range.

Marginal R^2^ values were < 0.29, indicating that latitude and species richness explained a relatively small amount of the total variation in cannibalism. These relatively weak relationships might be explained mainly by three factors: limitations of dietary data, undocumented influence of the evenness component of species diversity, and error in estimates of local species richness. Dietary analysis usually underestimates cannibalism, because food items rarely can be identified to species level, especially when material is partially digested. Furthermore, eggs and larvae, the stages most vulnerable to predation by conspecifics, are difficult to identify to species level and are digested even more rapidly than small fish. Despite these limitations, gut contents analysis remains the principal method used to reveal cannibalism. Future studies of cannibalisms could combine dietary analysis with more precise methods, such as DNA analysis. Additional dietary data from regions of the Southern Hemisphere that have even lower species richness might also reveal a stronger relationship. However, if the difference in the strength of relationship in the two hemispheres proves to be robust, this contrast should stimulate new theories and investigations. Another factor that should influence cannibalism is distribution of local prey abundance, because the evenness of this distribution should be just as important as species richness in determining encounter rates with conspecific versus heterospecific prey. In species-rich communities dominated by few abundant species, encounter rates with different prey will be skewed, with some prey more likely consumed than others [65]. Consequently, a numerically dominant species in a diverse community could still be prone to cannibalism. Finally, local fish species richness accounted only for fish as potential prey, and, given that most cannibals were not exclusively piscivorous, inclusion of non-fish prey might negate or reinforce the pattern. Nonetheless, fish species richness and total richness of aquatic animals should be correlated.

Despite limitations of the dataset for our meta-analysis, the results lend support for the hypothesis that cannibalism is more prevalent among fishes in regions of low species richness. The role of cannibalism as a density-dependent mechanism regulating population dynamics [7] should be greater in species-poor communities. Moreover, a cannibalism gradient would have important evolutionary consequences. For example, there should be stronger selection for behavioral and morphological adaptations to avoid cannibalism among piscivorous fishes from high northern latitudes. Examples of such adaptations include rapid growth of early life stages, development of dorsal spines, and habitat selection resulting in spatial segregation of vulnerable life stages from adults [66–69]. To more effectively test the hypothesis of a latitudinal gradient of cannibalism, there is the need to advance beyond tests of presence and absence of cannibalism. More detailed field studies that examine the intensity of cannibalism within populations at different locations are required.

Ricker considered cannibalism to be one of the most important mechanisms of fish population regulation [1,70]. Several empirical and experimental studies subsequently provided support for this idea, and inclusion of cannibalism sometimes can improve demographic models [71]. However, high levels of noise in population survey data and confounding effects of environmental variability make it difficult to demonstrate convincingly that cannibalism and other biotic interactions play significant roles in regulating fish stocks [72]. Future studies could evaluate additional variables, such as population density, body size distribution, and trophic guilds. Also, research is needed to document global patterns of fish species richness in the marine realm in order to test the relationship of cannibalism with species richness. Finally, given that human actions are causing biotic homogenization that decreases species diversity at local to regional scales [73–77], cannibalism might become more prevalent in the future, a possibility that merits investigation.

## Supporting Information

S1 Fig **Quantile-quantile plots (A), partial residual plots (B, C), and quantile-quantile plots of random effects (D) generated for the best models of each analyzed dataset.** Confidence intervals in the Quantile-quantile plots (A) were created based on the methodology proposed by Landwehr et al. (1984). Trend lines in panels B and C were modeled with LOESS. Details about the theory underling these plots and their interpretation are given by Zuur et al. (2009).
(PDF)

S1 Table Comparison of models with and without the random variable *genus* for each analyzed dataset using the Conditional Akaike Information Criterion (cAIC). The constant 1 included in all models refers to the intercept. The expression ‘(1|Genus)’ indicates the inclusion of random intercepts for fish genus.
(PDF)

S2 Table Number of cannibalism cases obtained in the literature search, with species feeding habit of the adult stage and countries where studies were conducted. ‘Total papers’ refers to the number of papers consulted for each species regardless of the presence or absence of cannibalism. Feeding habit: Inv- Invertivorous, Omn–Omnivorous, Pis–Piscivorous, and Zoo–Zooplanktivorous.
(PDF)

S3 Table PRISMA 2009 Checklist. The 27 checklist items pertain to the content of the systematic review and meta-analysis.
(DOC)

S4 Table PRISMA 2009 flow diagram. Number of records identified, included and excluded, and the reasons for exclusions for the systematic review and meta-analysis.
(DOC)
