# Supplementary material for: Is There a Relationship between Fish Cannibalism and Latitude or Species Richness?
Source: PLoS One. 2017 Jan 25;12(1):e0169813. doi: 10.1371/journal.pone.0169813 (PMC5266261; doi:10.1371/journal.pone.0169813)
Supplement: S1 Fig — Quantile-quantile plots (A), partial residual plots (B, C), and quantile-quantile plots of random effects (D) generated for the best models of each analyzed dataset. Confidence intervals in the Quantile-quantile plots (A) were created based on the methodology proposed by Landwehr et al. (1984). Trend lines in panels B and C were modeled with LOESS. Details about the theory underling these plots and their interpretation are given by Zuur et al. (2009). (PDF) [file pone.0169813.s001.pdf]

Pereira, L. S., Keppeler, F. W., Agostinho, A. A. and Winemiller, K. O. 2016. Is there a relationship between fish cannibalism and latitude or species richness?

S1 Fig. Quantile-quantile plots (A), partial residual plots (B,C), and quantile-quantile plots of random effects (D) generated for the best models of each dataset analyzed (Freshwater realm–Northern Hemisphere, Marine realm–Northern Hemisphere, Freshwater and Marine realms–Northern Hemisphere, Freshwater realm–Southern Hemisphere, Marine realm–Southern Hemisphere). Confidence intervals in the quantile-quantile plots (A) were created based on the methodology proposed by Landwehr et al. (1984). Trend lines in panels B and C were modelled with LOESS. Details about the theory underling these plots and their interpretation are given by Zuur et al. (1; 2009).

Freshwater North. Best model (Cannibalism occurrence ~ 1 + Latitude + Fish richness)

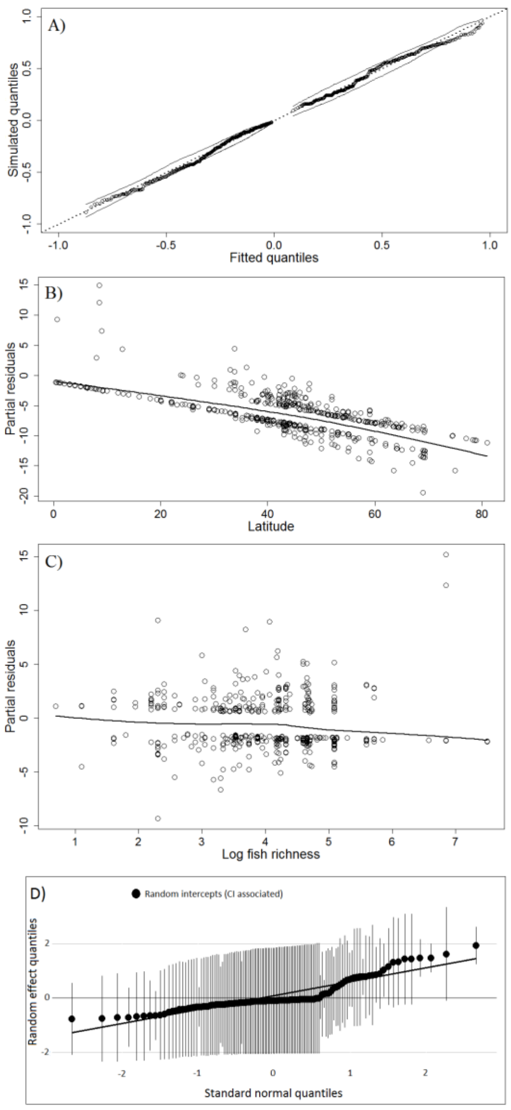

Freshwater North. Second best model (Cannibalism occurrence ~ 1 + Latitude)

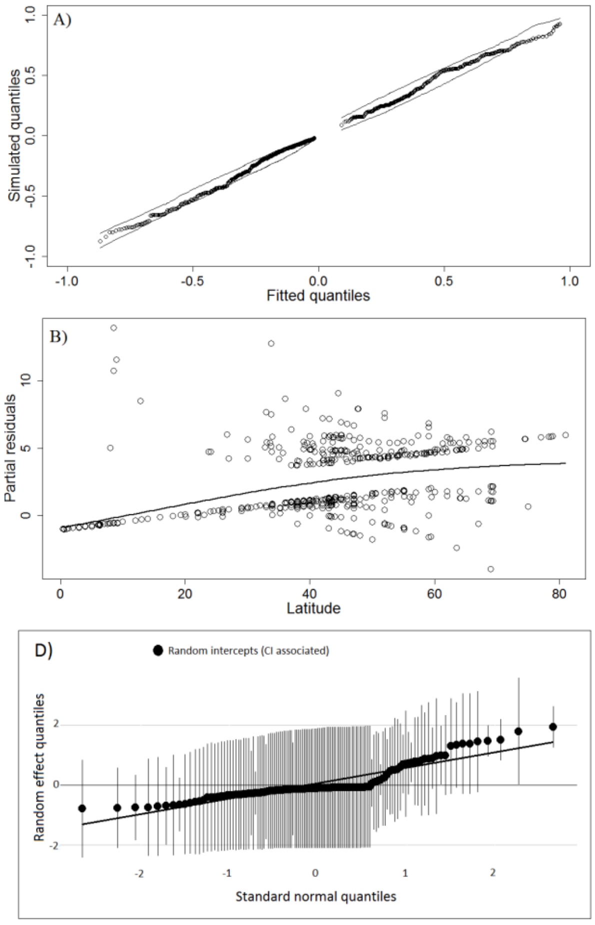

Pereira, L. S., Keppeler, F. W., Agostinho, A. A. and Winemiller, K. O. 2016. Is there a relationship between fish cannibalism and latitude or species richness?

Marine North. Best model (Cannibalism occurrence ~ 1 + Latitude)

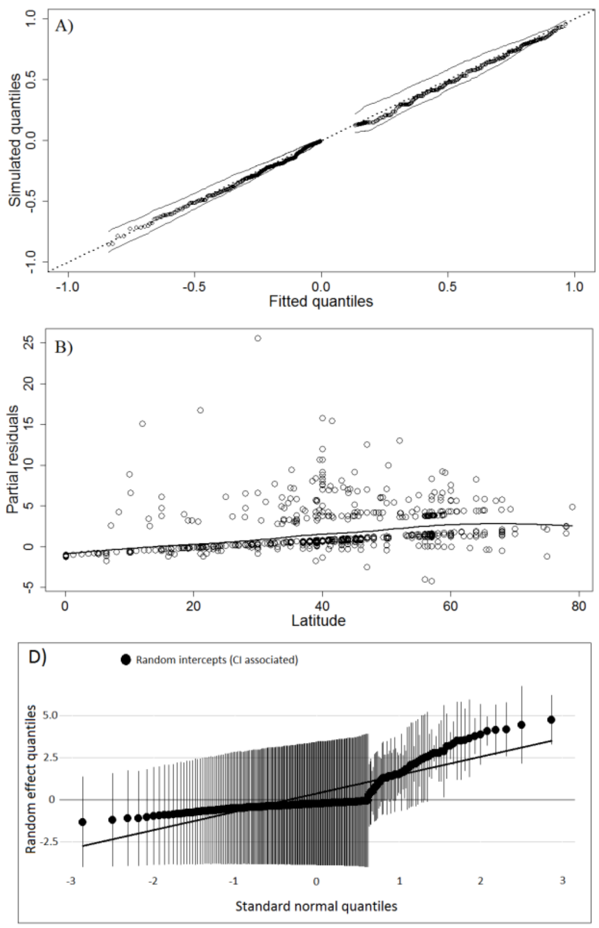

Northern hemisphere. Best model (Cannibalism occurrence ~ 1 + Latitude + Environment + Latitude*Environment)

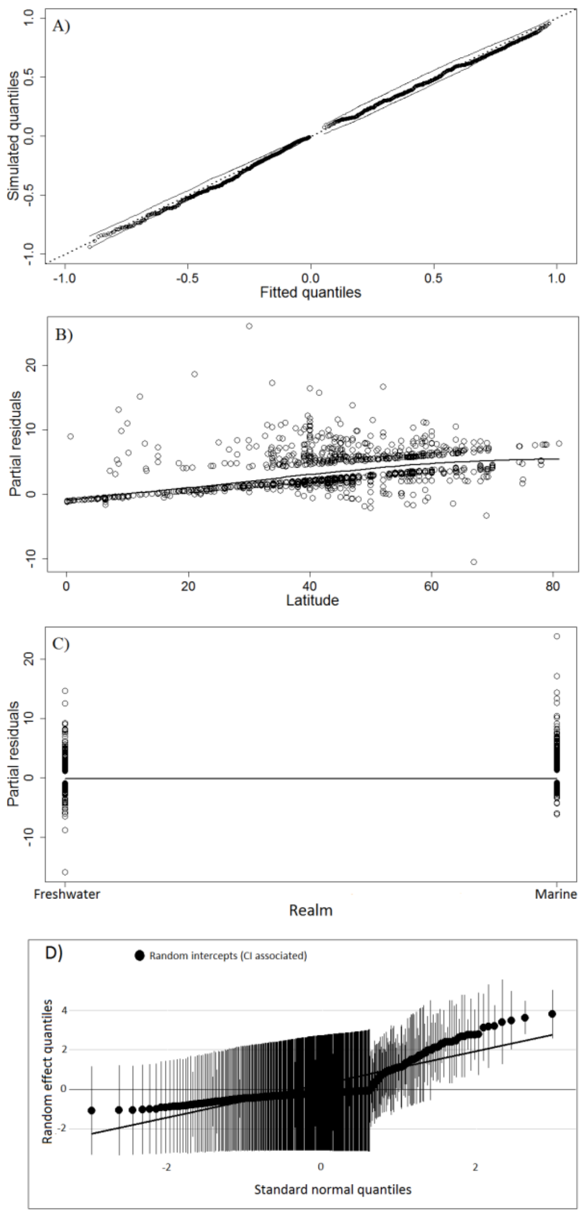

Pereira, L. S., Keppeler, F. W., Agostinho, A. A. and Winemiller, K. O. 2016. Is there a relationship between fish cannibalism and latitude or species richness?

Freshwater South. Best model (Cannibalism occurrence ~ 1 + Fish richness)

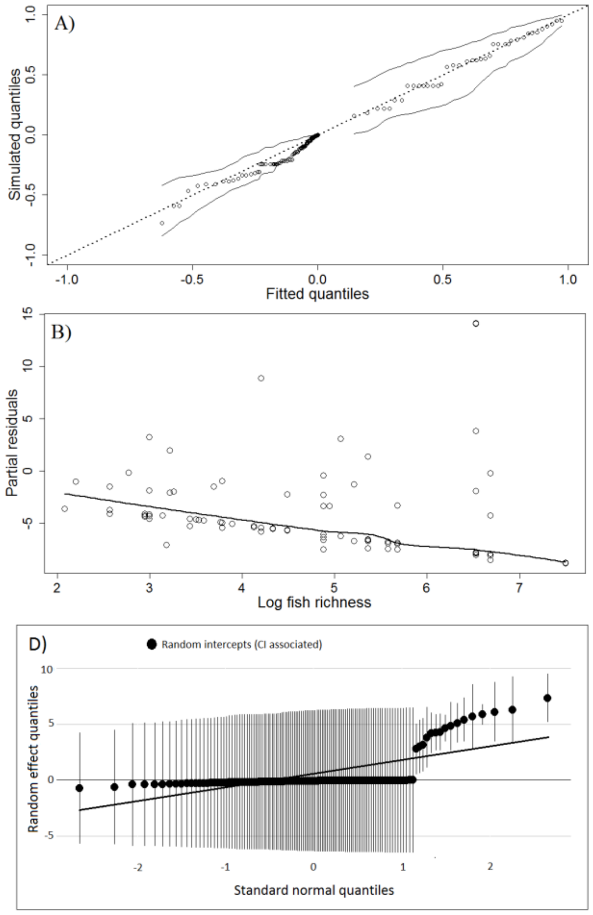

Freshwater South. Second best model (Cannibalism occurrence ~ Latitude + Fish richness)

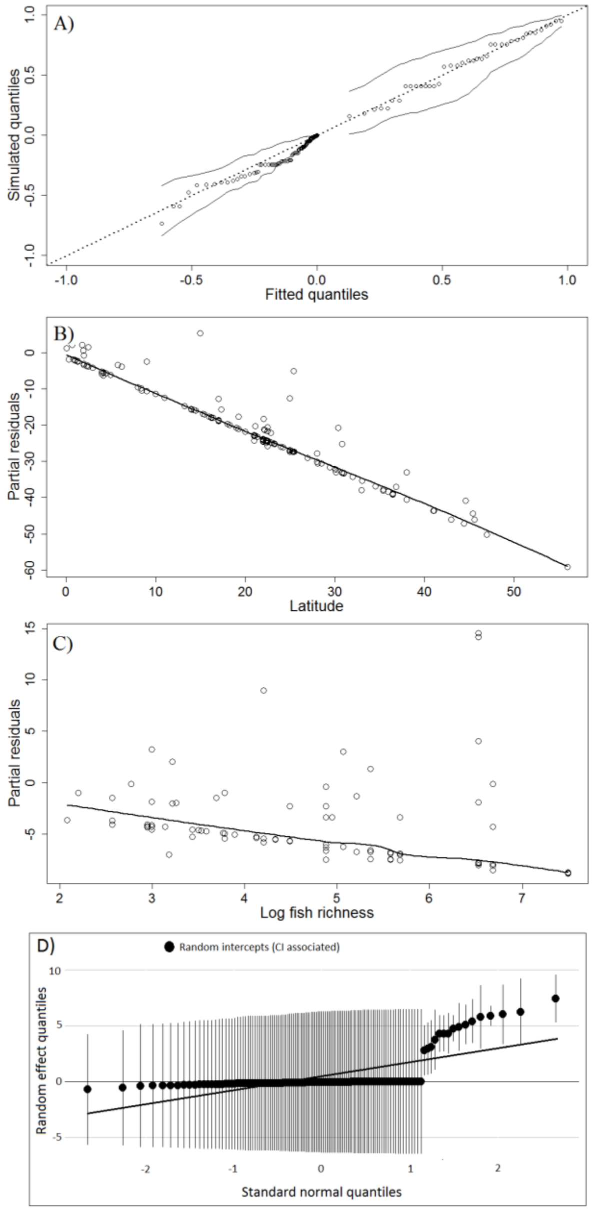

Pereira, L. S., Keppeler, F. W., Agostinho, A. A. and Winemiller, K. O. 2016. Is there a relationship between fish cannibalism and latitude or species richness?

Marine South. Best model (Cannibalism occurrence ~ 1 + Latitude)

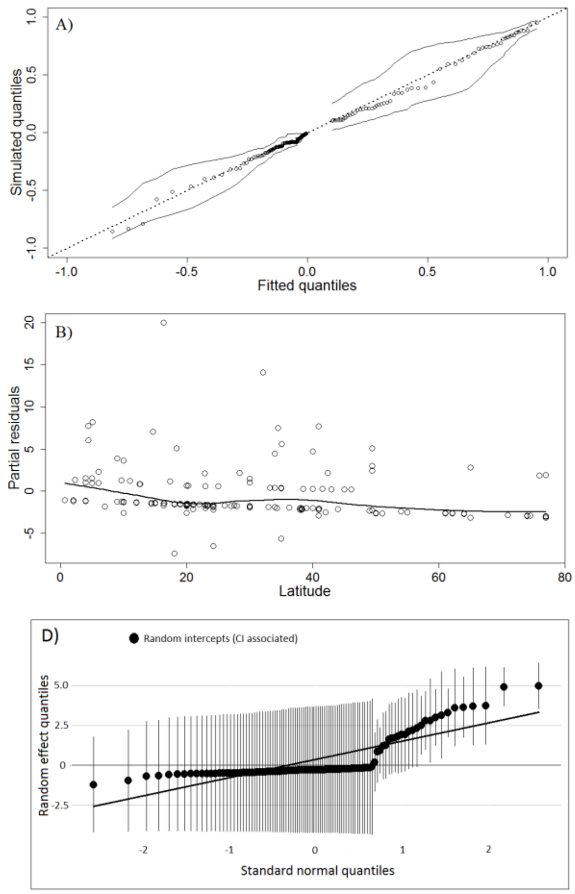

Marine South. Second best model (Cannibalism occurrence ~ 1)

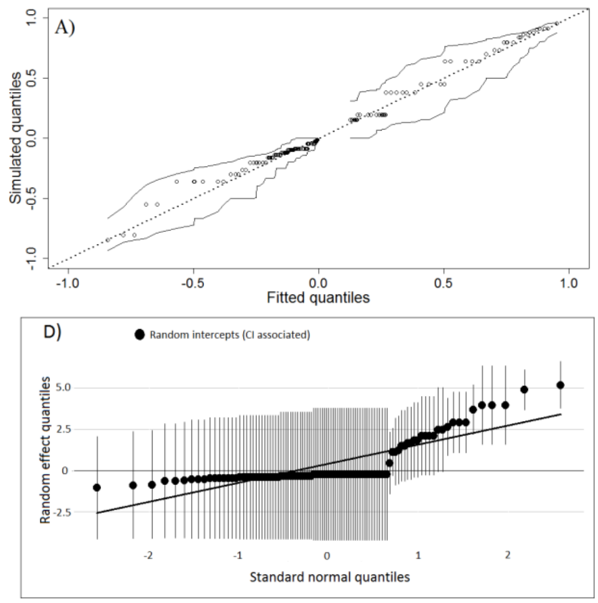

References

Landwehr JM, Pregibon D, Shoemaker AC. 1984. Graphical methods for assessing logistic regression models. Journal of the American Statistical Association 79:61–71

Zuur, A.F., Ieno, E.N., Walker,N.J., Saveliev, A.A. & Smith, G. 2009. Mixed Effects Models and Extensions in Ecology with R. Springer, New York.
