# Supplementary material for: Is There a Relationship between Fish Cannibalism and Latitude or Species Richness?
Source: PLoS One. 2017 Jan 25;12(1):e0169813. doi: 10.1371/journal.pone.0169813 (PMC5266261; doi:10.1371/journal.pone.0169813)
Supplement: S1 Table — The constant 1 included in all models refers to the intercept. The expression ‘(1|Genus)’ indicates the inclusion of random intercepts for fish genus. (PDF) [file pone.0169813.s002.pdf]

Pereira, L. S., Keppeler, F. W., Agostinho, A. A. and Winemiller, K. O. 2016. Is there a relationship between fish cannibalism and latitude or species richness?

S1 Table. Comparison of models with and without the random variable *genus* for each dataset based on the Conditional Akaike Information Criterion (cAIC; 1, 2). The constant 1 refers to the intercept; the expression '(1|Genus)' indicates the inclusion of random intercepts for *genus*.

Realm	Hemisphere	Rank	Model	cAIC
Freshwater	North	1°	1 + Latitude + Richness + (1|Genus)	1148.72
		2°	1 + Latitude + Richness	1178.61
	South	1°	1 + Latitude + Richness + (1|Genus)	251.19
		2°	1 + Latitude + Richness	316.98
Marine	North	1°	1 + Latitude + (1|Genus)	1202.05
		2°	1 + Latitude	1379.23
	South	1°	1 + Latitude + (1|Genus)	375.41
		2°	1 + Latitude	397.74
Freshwater and Marine	North	1°	1 + Latitude + Realm + Latitude*Realm + (1|Genus)	2372.57
		2°	1 + Latitude + Realm + Latitude*Realm	2584.71

References

1. Vaida F., Blanchard S. Conditional Akaike Information for Mixed-Effects Models. Biometrika. 2005; 92 (2): 351-370.
2. Saefken B., Ruegamer D., Greven S., Kneib T. cAIC4: Conditional Akaike information criterion for lme4. R package version 0.2. 2014. Available from: https://CRAN.R-project.org/package=cAIC4
