# Supplementary material for: Is There a Relationship between Fish Cannibalism and Latitude or Species Richness?
Source: PLoS One. 2017 Jan 25;12(1):e0169813. doi: 10.1371/journal.pone.0169813 (PMC5266261; doi:10.1371/journal.pone.0169813)
Supplement: S2 Table — ‘Total papers’ refers to the number of papers consulted for each species regardless of the presence or absence of cannibalism. Feeding habit: Inv- Invertivorous, Omn–Omnivorous, Pis–Piscivorous, and Zoo–Zooplanktivorous. (PDF) [file pone.0169813.s003.pdf]

Pereira, L. S., Keppeler, F. W., Agostinho, A. A. and Winemiller, K. O. 2016. Is there a relationship between fish cannibalism and latitude or species richness?

S2 Table. Number of cannibalism cases obtained in the literature search, with species feeding habit and countries where studies were conducted. Total papers refers to the number of papers consulted for each species despite the presence or absence of cannibalism. Feeding habit refers to the adult stage: Inv-Invertivorous, Omn – Omnivorous, Pis – Piscivorous, and Zoo – Zooplanktivorous.

Order	Family	Species	Habit	Total Papers	Cannibalism Cases	Environment	Country
Anguilliformes	Anguillidae	*Anguilla anguilla* [1] (Linnaeus, 1758)	Inv	16	1	Freshwater	Portugal
		Anguilla rostrata [2] (Lesueur, 1817)	Pis	2	1	-	USA
	Congridae	*Conger conger* [3–5] (Linnaeus, 1758)	Pis	7	3	Marine	Tunisia
		Conger oceanicus [2] (Mitchill, 1818)	Pis	1	1	-	USA
	Muraenesocidae	*Muraenesox cinereus* [6] (Forsskål, 1775)	Pis	1	1	Marine	Catamarans
Atheriniformes	Telmatherinidae	*Telmatherina sarasinorum* [7–9] Kottelat, 1991	Inv	5	3	Freshwater	Indonesia
		Telmatherina celebensis [7] Boulenger, 1897	Inv	1	1	Freshwater	Indonesia
Aulopiformes	Alepisauridae	*Alepisaurus ferox* [10–15] Lowe, 1833	Pis	9	6	Marine	Australia, Kenya, Seychelles
	Synodontidae	*Harpadon nehereus* [16,17] (Hamilton, 1822)	Pis	2	2	Marine	China
Batrachoidiformes	Batrachoididae	*Porichthys notatus* [18] Girard, 1854	Inv	2	1	Marine	Canada
Characiformes	Acestrorhynchidae	*Acestrorhynchus lacustris* [19] (Lütken, 1875)	Pis	6	1	Freshwater	Brazil
		Acestrorhynchus pantaneiro [20] Menezes, 1993	Pis	4	1	Freshwater	Brazil
	Distichodontidae	*Ichthyborus quadrilineatus* [21] (Pellegrin, 1904)	Pis	1	1	Freshwater	Congo
	Erythrinidae	*Hoplias malabaricus* [22,23] (Bloch, 1794)	Pis	30	2	Freshwater	Brazil
	Bryconidae	*Salminus brasiliensis* [24] (Cuvier, 1816)	Pis	11	2	Freshwater	Argentina

Clupeiformes	Clupeidae	*Alosa pseudoharengus* [25–28] (Wilson, 1811)	Zoo	11	4	Freshwater	Canada, USA
		Alosa sapidissima [29] (Wilson, 1811)	Omn	3	1	Freshwater	USA
		Clupea harengus [30–32] Linnaeus, 1758	Inv	25	3	Marine	Norway
		Jenkinsia lamprotaenia [33] (Gosse, 1851)	Inv	1	1	Marine	USA
		Limnothrissa miodon [34,35] (Boulenger, 1906)	Zoo	2	2	Freshwater	Rwanda
		Sardina pilchardus [36,37] (Walbaum, 1792)	Zoo	9	2	Marine	Portugal, Spain
		Sardinops sagax [38–40] (Jenyns, 1842)	Zoo	7	3	Marine	Japan, Peru, USA
		Sprattus sprattus [32,41,42] (Linnaeus, 1758)	Inv	13	3	Marine	Italy
	Engraulidae	*Encrasicholina devisi* [43] (Whitley, 1940)	Pis	1	1	Marine	New Guinea
		Encrasicholina heteroloba [43] (Rüppell, 1837)	Pis	1	1	Marine	New Guinea
		Engraulis anchoita [44,45] Hubbs & Marini, 1935	Zoo	2	2	Marine	Argentina
		Engraulis capensis [46–49] Gilchrist, 1913	Zoo	5	4	Marine	South Africa
		Engraulis encrasicolus [50,51] (Linnaeus, 1758)	Zoo	10	2	Marine	Greece, Spain
		Engraulis japonicus [52,53] Temminck & Schlegel, 1846	Zoo	5	2	Marine	Japan
		Engraulis ringens [38,39,54–57] Jenyns, 1842	Zoo	7	6	Marine	Chile, Peru
		Engraulis mordax [58–60] Girard, 1854	Zoo	3	1	Marine	USA
Cypriniformes	Catostomidae	*Moxostoma valenciennesi* [61] Jordan, 1885	Inv	1	1	Freshwater	Canada
	Cyprinidae	*Barbus mattozi* [62] Guimarães, 1884	Omn	1	1	Freshwater	Zimbabwe
		Carassius carassius [63] (Linnaeus, 1758)	Omn	1	1	Freshwater	Finland
		Nipponocypris temminckii [64–66] (Temminck & Schlegel, 1846)	Omn	4	3	Freshwater	Japan
(…)	(…)	*Ptychocheilus grandis* [67,68] (Ayres, 1854)	Omn	3	2	Freshwater	USA

(…)	(…)	*Semotilus atromaculatus* [69] (Mitchill, 1818)	Omn	4	1	Freshwater	-
Cypriniformes	Cyprinidae	*Squalius pyrenaicus* [70] (Günther, 1868)	Pis	1	1	Marine	Portugal
		Zacco platypus [71] (Temminck & Schlegel, 1846)	Inv	1	1	Freshwater	Japan
		Rutilus rutilus [72] (Linnaeus, 1758)	Zoo	7	1	Freshwater	Germany
Cyprinodontiformes	Fundulidae	*Fundulus heteroclitus* [73] (Linnaeus, 1766)	Pis	4	1	Freshwater	USA
	Poeciliidae	*Gambusia affinis* [74] (Baird & Girard, 1853)	Inv	8	1	Freshwater	Turkey
		Gambusia holbrooki [75–77] Girard, 1859	Inv	6	3	Freshwater	Australia, Hungary, Australis
Esociformes	Esocidae	*Esox lucius* [78–106] Linnaeus, 1758	Inv-Pis	61	30	Freshwater	Canada, Denmark, Englad, Finland, France, Italy, Netherlands, Russia, Sweden, Turkey, USA
	Umbridae	*Umbra pygmaea* [107] (DeKay, 1842)	Inv	1	1	Freshwater	Croatia
Gadiformes	Gadidae	*Gadus macrocephalus* [108] Tilesius, 1810	Pis	8	1	Marine	Canada
		Gadus morhua [32,109–144] Linnaeus, 1758	Inv	82	41	Marine	Canada, Norway, Russia, UK, USA
		Melanogrammus aeglefinus [116,145] (Linnaeus, 1758)	Zoo	9	2	Marine	UK, USA
		Merlangius merlangus [134,146,147] (Linnaeus, 1758)	Pis	10	3	Marine	Norway, UK
		Micromesistius poutassou [148] (Risso, 1827)	Inv	7	1	Marine	Portugal
		Pollachius pollachius [129,134] (Linnaeus, 1758)	Inv	2	2	Marine	Norway
		Pollachius virens [145,147] (Linnaeus, 1758)	Inv	10	2	Marine	-
		Theragra chalcogramma [108,149–177] (Pallas, 1814)	Pis	36	30	Marine	Alaska, Canada, Japan, Russia
		Trisopterus esmarkii [116] (Nilsson, 1855)	Zoo	2	1	Marine	UK
(…)	Lotidae	*Lota lota* [178–180] (Linnaeus, 1758)	Pis	8	3	Freshwater	Canada

(…)	Merlucciidae	*Macruronus novaezelandiae* [181,182] (Hector, 1871)	Pis	4	2	Marine	Tasmania
Gadiformes	Merlucciidae	*Merluccius albidus* [183] (Mitchill, 1818)	Pis	1	1	Marine	USA
		Merluccius bilinearis [131,145,183–185] (Mitchill, 1814)	Pis	8	5	Marine	Canada, USA
		Merluccius capensis [186–188] Castelnau, 1861	Pis	3	3	Marine	Angola, Namibia, South Africa
		Merluccius gayi [189–194] (Guichenot, 1848)	Inv	6	6	Marine	Chile, Peru
		Merluccius hubbsi [195–198] Marini, 1933	Pis	5	4	Marine	Argentina
		Merluccius merluccius [148,199–203] (Linnaeus, 1758)	Pis	14	6	Marine	France, Portugal, Spain
		Merluccius productus [204] (Ayres, 1855)	Pis	2	1	Marine	USA
	Phycidae	*Urophycis brasiliensis* [205] (Kaup, 1858)	Inv	3	1	Marine	Uruguay
		Urophycis chuss [145,183] (Walbaum, 1792)	Pis	4	2	Marine	USA
		Urophycis regia [145,183] (Walbaum, 1792)	Inv	4	2	Marine	USA
		Urophycis tenuis [128,145,183] (Mitchill, 1814)	Pis	5	3	Marine	Canada
	Macrouridae	*Lepidorhynchus denticulatus* [181] Richardson, 1846	Omn	2	1	Marine	Tasmania
Gasterosteiformes	Gasterosteidae	*Gasterosteus aculeatus* [206–220] Linnaeus, 1758	Inv	37	16	Freshwater	Alaska, Canada, Finland, Japan, USA
		Gasterosteus wheatlandi [208] Putnam, 1867	Inv	1	1	Marine	Canada
Gobiesociformes	Gobiesocidae	*Diademichthys lineatus* [221] (Sauvage, 1883)	Inv	1	1	Marine	Japan
Lepidosireniformes	Protopteridae	*Protopterus aethiopicus* [222] Heckel, 1851	Pis	1	1	Freshwater	Kenya
	Lepisosteidae	*Lepisosteus osseus* [223] (Linnaeus, 1758)	Pis	3	1	Marine	USA
Lophiiformes	Lophiidae	*Lophius americanus* [145,224] Valenciennes, 1837	Pis	3	2	Marine	-
		Lophius piscatorius [225] Linnaeus, 1758	Pis	4	1	Marine	UK
Myctophiformes	Myctophidae	*Benthosema glaciale* [226] (Reinhardt, 1837)	Zoo	2	1	Marine	Mallorca Islands
		Lampanyctus crocodilus [226] (Risso, 1810)	Inv	1	1	Marine	Mallorca Islands

Osmeriformes	Galaxiidae	*Galaxias platei* [227] Steindachner, 1898	Inv	1	1	Freshwater	Chile
		Neochanna burrowsius [228] (Phillipps, 1926)	Inv	1	1	Freshwater	New Zealand
	Osmeridae	*Mallotus villosus* [229–231] (Müller, 1776)	Inv-Pis	9	3	Marine	Norway
		Osmerus eperlanus [232,233] (Linnaeus, 1758)	Inv	3	2	Freshwater	Finland, Ireland
		Osmerus mordax [234–240] (Mitchill, 1814)	Inv-Pis	13	7	Freshwater	Canada, USA
Perciformes	Ammodytidae	*Ammodytes dubius* [241] Reinhardt, 1837	Zoo	2	1	Marine	USA
		Ammodytes marinus [242] Raitt, 1934	Zoo	3	1	Marine	-
		Ammodytes personatus [243] Girard, 1856	Zoo	1	1	Marine	Japan
	Apogonidae	*Apogon imberbis* [244,245] (Linnaeus, 1758)	Inv	2	2	Marine	Italy, Spain
		Jaydia lineata [246–250] (Temminck & Schlegel, 1842)	Inv	6	5	Marine	Japan
		Ostorhinchus cyanosoma [251] (Bleeker, 1853)	Inv	2	1	Marine	Australia
		Ostorhinchus doederleini [252–256] (Jordan & Snyder, 1901)	Zoo	7	5	Marine	Japan
		Ostorhinchus notatus [257–259] (Houttuyn, 1782)	Inv	4	3	Marine	Japan
		Pterapogon kauderni [260] Koumans, 1933	Zoo	1	1	Marine	Indonesia
	Bathydraconidae	*Cygnodraco mawsoni* [261] Waite, 1916	Pis	1	1	Marine	Antarctica
		Gymnodraco acuticeps [261] Boulenger, 1902	Pis	2	1	Marine	Antarctica
	Centropomidae	*Centropomus undecimalis* [262,263] (Bloch, 1792)	Pis	3	2	Marine	USA
	Champsodontidae	*Champsodon snyderi* [264] Franz, 1910	Inv	1	1	Marine	Japan
	Blenniidae	*Aidablennius sphynx* [265] (Valenciennes, 1836)	Inv	1	1	Marine	France
		Salaria fluviatilis [266–268] (Asso, 1801)	Inv	4	2	Freshwater	Israel, Spain
(…)		*Salaria pavo* [269] (Risso, 1810)	Inv	2	1	Freshwater	Portugal

(…)		*Pomoxis nigromaculatus* [270]<br>(Lesueur, 1829)	Inv	5	1	Marine	USA
		Lepomis gibbosus [271]<br>(Linnaeus, 1758)	Inv	4	1	Freshwater	UK
		Lepomis macrochirus [272]<br>Rafinesque, 1819	Inv	16	1	Freshwater	Canada
	Centrarchidae	*Micropterus dolomieu* [273–275]<br>Lacepède, 1802	Inv-Pis	9	3	Freshwater	USA
		Micropterus salmoides [273,274,276–286]<br>(Lacepède, 1802)	Inv-Pis	34	13	Freshwater	Kenya, Mozambique, USA
		Pomoxis nigromaculatus [270]<br>(Lesueur, 1829)	Inv	1	1	Marine	USA
		Cichla kelberi [287,288]<br>Kullander & Ferreira, 2006	Pis	7	2	Freshwater	Brazil
		Cichla monoculus [289–292]<br>Agassiz, 1831	Pis	9	4	Freshwater	Brazil
		Cichla ocellaris [289,293,294]<br>Bloch & Schneider, 1801	Pis	4	3	Freshwater	Brazil
		Cichla piquiti [295]<br>Kullander & Ferreira, 2006	Pis	2	1	Freshwater	Brazil
Perciformes	Cichlidae	*Etroplus maculatus* [296]<br>(Bloch, 1795)	Inv	1	1	Freshwater	Sri Lanka
		Etroplus suratensis [296]<br>(Bloch, 1790)	Inv	1	1	Freshwater	Sri Lanka
		Lamprologus callipterus [297,298]<br>Boulenger, 1906	Inv	3	2	Freshwater	Congo
		Neolamprologus pulcher [299]<br>(Trewavas & Poll, 1952)	Zoo	3	1	Freshwater	Congo
		Oreochromis mossambicus [300]<br>(Peters, 1852)	Omn	7	1	Freshwater	South Africa
	Coryphaenidae	*Coryphaena hippurus* [10,301,302]<br>Linnaeus, 1758	Pis	10	3	Marine	Mexico
		Eleotris amblyopsis [22,303]<br>(Cope, 1871)	Inv-Pis	2	2	Marine	Costa Rica
	Eleotridae	*Gobiomorphus breviceps* [304]<br>(Stokell, 1939)	Inv	1	1	Freshwater	New Zealand
		Gobiomorus dormitor [305]<br>Lacepède, 1800	Pis	3	1	Freshwater	Nicaragua
	Gobiidae	*Bathygobius fuscus* [306]<br>(Rüppell, 1830)	Omn	1	1	Marine	Japan
		Glossogobius giuris [307,308]<br>(Hamilton, 1822)	Pis	3	2	Freshwater	Bangladesh, India
(…)	(…)	*Istigobius goldmanni* [309]<br>(Bleeker, 1852)	Omn	1	1	Marine	Australia

(…)	(…)	*Pomatoschistus marmoratus* [310] (Risso, 1810)	Inv	1	1	Marine	Italy
		Pomatoschistus minutus [311,312] (Pallas, 1770)	Inv	26	2	Marine	France, Norway
	Gobiidae	*Ponticola kessleri* [313] (Günther, 1861)	Omn	1	1	Freshwater	Germany
		Proterorhinus semilunaris [314] (Heckel, 1837)	Inv	1	1	Freshwater	Czech Republic
		Rhinogobius sp. [315,316]	Inv	6	2	Freshwater	Japan
	Labridae	*Symphodus ocellatus* [317] (Linnaeus, 1758)	Inv	1	1	Marine	France
		Xyrichtys martinicensis [318] Valenciennes, 1840	Inv	1	1	Marine	Caribbean Island of Bonaire
		Xyrichtys novacula [318] (Linnaeus, 1758)	Inv	2	1	Marine	Caribbean Island of Bonaire
		Xyrichtys splendens [318] Castelnau, 1855	Inv	1	1	Marine	Caribbean Island of Bonaire
Perciformes	Latidae	*Lates calcarifer* [319] (Bloch, 1790)	Pis	17	1	Marine	Australia
		Lates niloticus [320–322] (Linnaeus, 1758)	Pis	4	3	Freshwater	Ethiopia, Rwanda, Uganda
	Lutjanidae	*Lutjanus campechanus* [323] (Poey, 1860)	Pis	6	1	Marine	-
	Moronidae	*Dicentrarchus labrax* [324] (Linnaeus, 1758)	Pis	18	1	Both	UK
		Morone americana [325,326] (Gmelin, 1789)	Pis	5	2	Freshwater	USA
		Morone chrysops [326] (Rafinesque, 1820)	Pis	4	1	Freshwater	USA
		Morone saxatilis [327] (Walbaum, 1792)	Pis	13	1	Marine	USA
	Nemipteridae	*Nemipterus japonicus* [328] (Bloch, 1791)	Pis	2	1	Marine	India
	Nototheniidae	*Dissostichus eleginoides* [329] Smitt, 1898	Pis	2	1	Marine	Chile
		Pleuragramma antarctica [330,331] Boulenger, 1902	Pis	4	2	Marine	Antarctica
	Odontobutidae	*Perccottus glenii* [332] Dybowski, 1877	Inv	2	1	Freshwater	Slovakia
(…)	Percichthyidae	*Percichthys colhuapiensis* [333] MacDonagh, 1955	Inv	1	1	Freshwater	Argentina

(…)	Percidae	*Etheostoma olmstedi* [334] Storer, 1842	Inv	1	1	Freshwater	USA
		Perca flavescens [274,326,335–339] (Mitchill, 1814)	Inv-Pis	17	7	Freshwater	USA
		Perca fluviatilis [72,79,91,93,99,102,340–369] Linnaeus, 1758	Inv-Pis	76	36	Freshwater	Czech Republic, Finland, Germany, Hungary, Italy, Poland, Sweden, Turkey, Ukraine, UK, USA
		Sander canadensis [370] (Griffith & Smith, 1834)	Pis	1	1	Freshwater	USA
		Sander lucioperca [353,355,357,371–386] (Linnaeus, 1758)	Pis	33	19	Freshwater	Estonia, Germany, Finland, France, Netherlands, Poland, Spain, Turkey
		Sander vitreus [270,326,335,387–394] (Mitchill, 1818)	Pis	30	11	Freshwater	Canada, USA
		Sander volgensis [395] (Gmelin, 1789)	Pis	2	1	Freshwater	Hungary
Perciformes	Pomacentridae	*Abudefduf saxatilis* [396–398] (Linnaeus, 1758)	Zoo	3	3	Marine	Barbados
		Chromis notata [399] (Temminck & Schlegel, 1843)	Zoo	1	1	Marine	Japan
		Hypsypops rubicundus [400] (Girard, 1854)	Inv	2	1	Marine	USA
		Pomacentrus amboinensis [401] Bleeker, 1868	Omn	1	1	Marine	Australia
		Pomacentrus nagasakiensis [399] Tanaka, 1917	Omn	1	1	Marine	Japan
		Stegastes leucostictus [402] (Müller & Troschel, 1848)	Omn	4	1	Marine	Jamaica
		Stegastes rectifraenum [403–405] (Gill, 1862)	Omn	4	3	Marine	Canada, USA
	Pomatomidae	*Pomatomus saltatrix* [145,406] (Linnaeus, 1766)	Pis	14	2	Marine	USA
	Sphyraenidae	*Sphyraena guachancho* [407] Cuvier, 1829	Pis	1	1	Marine	Ivory Coast
	Sciaenidae	*Cynoscion regalis* [241,408] (Bloch & Schneider, 1801)	Pis	6	2	Marine	USA
		Macrodon ancylodon [429, 498] (Bloch & Schneider, 1801)	Inv	2	2	Marine	Brazil
		Micropogonias furnieri [409] (Desmarest, 1823)	Inv	4	1	Marine	Argentina
		Plagioscion squamosissimus [410,411] (Heckel, 1840)	Inv-Pis	15	2	Freshwater	Brazil
(…)	Scombridae	*Katsuwonus pelamis* [10,412,413] (Linnaeus, 1758)	Pis	7	3	Marine	Japan

(…)	(…)	*Thunnus alalunga* [414] (Bonnaterre, 1788)	Inv-Pis	10	1	Marine	USA
	Scombridae	*Thunnus maccoyii* [415] (Castelnau, 1872)	Pis	2	1	Marine	-
		Thunnus orientalis [416] (Temminck & Schlegel, 1844)	Pis	6	1	Marine	Spain
		Scomber scombrus [417–421] Linnaeus, 1758	Pis	14	5	Marine	Canada, UK, USA
Perciformes	Serranidae	*Epinephelus malabaricus* [422] (Bloch & Schneider, 1801)	Inv-Pis	5	1	Marine	Brazil
		Epinephelus merra [423] Bloch, 1793	Pis	4	1	Marine	Reunion Island
	Trichiuridae	*Lepidopus caudatus* [424] (Euphrasen, 1788)	Inv	3	1	Marine	-
		Trichiurus lepturus [425–432] Linnaeus, 1758	Inv-Pis	13	8	Marine	Brazil, China, Japan, Mexico
		Trichiurus margarites [433] Li, 1992	Pis	2	1	Marine	China
	Tripterygiidae	*Enneapterygius etheostomus* [434,435] (Jordan & Snyder, 1902)	Inv	2	2	Marine	Australia
Pleuronectiformes	Paralichthyidae	*Citharichthys spilopterus* [436] Günther, 1862	Inv	3	1	Freshwater	Venezuela
		Paralichthys olivaceus [437–440] (Temminck & Schlegel, 1846)	Pis	11	4	Marine	Japan
		Paralichthys orbignyanus [441] (Valenciennes, 1839)	Pis	2	1	Marine	Uruguay
	Pleuronectidae	*Platichthys flesus* [442] (Linnaeus, 1758)	Inv	7	1	Marine	France
		Reinhardtius hippoglossoides [443–445] (Walbaum, 1792)	Pis	9	3	Marine	Canada, Greenland
(…)		*Hippoglossus stenolepis* [446] Schmidt, 1904	Pis	3	1	Marine	Alaska, Canada
(…) Pleuronectiformes	Scophthalmidae	*Scophthalmus aquosus* [241,447] (Mitchill, 1815)	Zoo	6	2	Marine	Canada
Salmoniformes	Salmonidae	*Coregonus artedi* [448] Lesueur, 1818	Zoo	2	1	Freshwater	USA
		Coregonus clupeaformis [89] (Mitchill, 1818)	Inv	5	1	Freshwater	Russia
		Coregonus lavaretus [449–451] (Linnaeus, 1758)	Zoo	8	3	Freshwater	Germany, Norway
		Oncorhynchus gilae [452,453] (Miller, 1950)	Inv	2	2	Freshwater	USA
		Oncorhynchus gorbuscha [454–456] (Walbaum, 1792)	Pis	7	3	Freshwater	Canada, Russia, USA

		Oncorhynchus kisutch [457] (Walbaum, 1792)	Pis	5	1	Freshwater	USA
		Oncorhynchus mykiss [458–460] (Walbaum, 1792)	Inv	19	3	Freshwater	Canada, China, New Zealand, USA
		Parahucho perryi [461] (Brevoort, 1856)	Inv	1	1	Freshwater	Japan
		Salmo salar [26,462] Linnaeus, 1758	Pis	9	2	Freshwater	Canada
		Salmo trutta [463–470] Linnaeus, 1758	Inv	35	8	Freshwater	France, Iran, Norway, UK
		Salvelinus alpinus [91,471–484] (Linnaeus, 1758)	Inv	48	22	Freshwater	Canada, Greenland, Norway, Russia, USA, UK, Sweden
		Salvelinus confluentus [485,486] (Suckley, 1859)	Pis	2	2	Freshwater	Canada
		Salvelinus fontinalis [487] (Mitchill, 1814)	Pis	5	1	Freshwater	Canada
		Salvelinus malma [488,489] (Walbaum, 1792)	Pis	4	2	Freshwater	Japan
		Salvelinus namaycush [457,490–494] (Walbaum, 1792)	Pis	11	6	Freshwater	Canada, USA
		Thymallus arcticus [495] (Pallas, 1776)	Inv	1	1	Freshwater	China
Scorpaeniformes	Cottidae	*Cottus carolinae* [496] (Gill, 1861)	Inv	1	1	Freshwater	USA
		Cottus gobio [497,498] Linnaeus, 1758	Inv	2	2	Freshwater	Italy
		Cottus nozawae [499] Snyder, 1911	Inv	1	1	Freshwater	Japan
		Cottus paulus [500] Williams, 2000	Inv	2	1	Freshwater	-
(…)	(…)	*Cottus perifretum* [501] Freyhof, Kottelat & Nolte, 2005	Inv	1	1	Freshwater	Belgium
(…) Scorpaeniformes	(…) Cottidae	*Myoxocephalus brandtii* [502] (Steindachner, 1867)	Inv	1	1	Marine	Japan
		Myoxocephalus octodecemspinosus [241] (Mitchill, 1814)	Inv	3	1	Marine	USA
		Myoxocephalus scorpius [503] (Linnaeus, 1758)	Inv	5	1	Marine	Iceland
		Myoxocephalus thompsonii [504] (Girard, 1851)	Inv	3	1	Freshwater	USA
	Hexagrammidae	*Hexagrammos otakii* [505] Jordan & Starks, 1895	Pis	3	1	Marine	Japan
		Ophiodon elongatus [506] Girard, 1854	Pis	1	1	Marine	Canada

		Oxylebius pictus [507] Gill, 1862	Inv	1	1	Marine	USA
		Pleurogrammus monopterygius [508–510] (Pallas, 1810)	Pis	4	3	Marine	Alaska, Russia
	Scorpaenidae	*Pterois volitans* [511] (Linnaeus, 1758)	Inv	3	1	Marine	Israel, Mexico
	Sebastidae	*Helicolenus percoides* [181] (Richardson & Solander, 1842)	Omn	2	1	Marine	Tasmania
		Sebastes fasciatus [241] Storer, 1854	Zoo	1	1	Marine	USA
		Sebastes norvegicus [120] (Ascanius, 1772)	Pis	2	1	Marine	Canada
	Hemitripteridae	*Hemitripterus americanus* [241] (Gmelin, 1789)	Pis	3	1	Marine	USA
Stomiiformes	Sternoptychidae	*Argyropelecus aculeatus* [512] Valenciennes, 1850	Zoo	1	1	Marine	Portugal
Siluriformes	Bagridae	*Rita rita* [513] (Hamilton, 1822)	Pis	1	1	Freshwater	Pakistan
		Sperata seenghala [513] (Sykes, 1839)	Pis	1	1	Freshwater	Pakistan
	Clariidae	*Clarias gariepinus* [514,515] (Burchell, 1822)	Omn-Pis	24	2	Freshwater	Botswania
		Clarias ngamensis [515] Castelnau, 1861	Omn-Pis	1	1	Freshwater	Botswania
	Ictaluridae	*Pylodictis olivaris* [516] (Rafinesque, 1818)	Inv-Pis	2	1	Freshwater	USA
	Schilbeidae	*Clupisoma garua* [513] (Hamilton, 1822)	Pis	1	1	Freshwater	Pakistan
		Clupisoma naziri [513] Mirza & Awan, 1973	Pis	1	1	Freshwater	Pakistan
(…)		*Schilbe intermedius* [515] Rüppell, 1832	Pis	2	1	Freshwater	Botswania
(…) Siluriformes	Siluridae	*Ompok bimaculatus* [513] (Bloch, 1794)	Inv-Pis	2	1	Freshwater	Pakistan
		Ompok pabda [513] (Hamilton, 1822)	Inv-Pis	1	1	Freshwater	Pakistan
		Silurus glanis [517,518] Linnaeus, 1758	Pis	6	2	Freshwater	Turckey
		Wallago attu [513] (Bloch & Schneider, 1801)	Pis	4	1	Freshwater	Pakistan
	Sisoridae	*Bagarius bagarius* [513] (Hamilton, 1822)	Pis	1	1	Freshwater	Pakistan
Syngnathiformes	Syngnathidae	*Hippocampus guttulatus* [519] Cuvier, 1829	Inv	1	1	Marine	Greece

		Hippocampus hippocampus [519] (Linnaeus, 1758)	Inv	1	1	Marine	Greece
		Hippocampus patagonicus [520] Piacentino & Luzzatto, 2004	Inv	1	1	Marine	Argentina
		Syngnathus floridae [521] (Jordan & Gilbert, 1882)	Inv	3	1	Marine	USA
		Syngnathus fuscus [521] Storer, 1839	Zoo	1	1	Marine	USA
		Syngnathus typhle [522] Linnaeus, 1758	Inv	1	1	Marine	Italy
Tetraodontiformes	Monacanthidae	*Paramonacanthus japonicus* [523] (Tilesius, 1809)	Inv	1	1	Marine	Japan
Zeiformes	Zeidae	*Zenopsis conchifer* [241] (Lowe, 1852)	Pis	1	1	Marine	USA

References

1. Domingos I, Costa JL, Costa MJ. Factors determining length distribution and abundance of the European eel, *Anguilla anguilla*, in the River Mondego (Portugal). Freshw Biol. 2006;51: 2265–2281.

2. Musumeci VL, Able KW, Sullivan MC, Smith JM. Estuarine predator—prey interactions in the early life history of two eels (*Anguilla rostrata* and *Conger oceanicus*). Environ Biol Fishes. 2014;97: 929–938.

3. Sallami B, Ben Salem M, Reynaud C. Diet of European conger eel *Conger conger* (Osteichthyes: Congridae) from the northeastern coast of Tunisia (central Mediterranean). Cah Biol Mar. 2015;56: 253–262.

4. Xavier JC, Cherel Y, Assis CA, Sendão J, Borges TC. Feeding ecology of conger eels (*Conger conger*) in north-east Atlantic waters. J Mar Biol Assoc United Kingdom. 2010;90: 493–501.

5. Cau A, Manconi P. Relationship of feeding, reproductive cycle and bathymetric distribution in *Conger conger*. Mar Biol. 1984;81: 147–151.

6. Devadoss P, Pillai PK. Observations on the food and feeding habits of the eel, *Muraenesox cinereus* (Forskal) from Porto Novo. Indian J Fish. 1979;26: 244–246.

7. Gray SM, McKinnon JS. A comparative description of mating behaviour in the endemic telmatherinid fishes of Sulawesi’s Malili Lakes. Environ Biol Fishes. 2006;75: 471–482.

8. Gray SM, Dill LM, McKinnon JS. Cuckoldry incites cannibalism: male fish turn to cannibalism when perceived certainty of paternity decreases. Am Nat. 2007;169: 258–263. doi:10.1086/510604

9. Gray SM, McKinnon JS, Tantu FY, Dill LM. Sneaky egg-eating in *Telmatherina sarasinorum*, an endemic fish from Sulawesi. J Fish Biol. 2008;73: 728–731.

10. Varghese SP, Somvanshi VS, Dalvi RS. Diet composition, feeding niche partitioning and trophic organisation of large pelagic predatory fishes in the eastern Arabian Sea. Hydrobiologia. 2014;736: 99–114.

11. Young JW, Lansdell MJ, Campbell RA, Cooper SP, Juanes F, Guest MA. Feeding ecology and niche segregation in oceanic top predators off eastern Australia. Mar Biol. 2010;157: 2347–2368.

12. Potier M, Marsac F, Cherel Y, Lucas V, Sabatié R, Maury O, et al. Forage fauna in the diet of three large pelagic fishes (lancetfish, swordfish and yellowfin tuna) in the western equatorial Indian Ocean. Fish Res. 2007;83: 60–72.

13. Romanov E V., Zamorov V V. Regional feeding patterns of the longnose lancetfish (*Alepisaurus ferox* Lowe, 1833) of the western Indian Ocean. J Mar Sci. 2007;6: 1–37.

14. Potier M, Menard F, Cherel Y, Lorrain A, Sabatié R, Marsac F. Role of pelagic crustaceans in the diet of the longnose lancetfish *Alepisaurus ferox* in the Seychelles waters. African J Mar Sci. 2007;29: 113–122.

15. Romanov E V., Ménard F, Zamorov V V., Potier M. Variability in conspecific predation among longnose lancetfish *Alepisaurus ferox* in the western Indian Ocean. Fish Sci. 2008;74: 62–68. doi:10.1111/j.1444-2906.2007.01496.x

16. Lin XP, Zhu ZJ, Li PF. Feeding habits of *Harpadon nehereus* in the East China Sea region. Mar Fish. 2010;32: 290–296.

17. Zhang B, Jin X. Feeding habits and ontogenetic diet shifts of Bombay duck, *Harpadon nehereus*. Chinese J Oceanol Limnol. 2014;32: 542–548.

18. Cogliati KM, Danukarjanto C, Pereira AC, Lau MJ, Hassan A, Mistakidis AF, et al. Diet and cannibalism in plainfin midshipman *Porichthys notatus*. J Fish Biol. 2015;86: 1396–1415.

19. da Rocha AAF, dos Santos NLC, de Araújo Pinto G, do Nascimento Medeiros T, Severi W. Diet composition and food overlap of *Acestrorhynchus britskii* and *A. lacustris* (Characiformes: Acestrorhynchidae) from Sobradinho reservoir, São Francisco river, Bahia State. Acta Sci Biol Sci. 2011;33: 407–415.

20. Krinski D. Dieta do peixe-cachorro Acestrorhynchus pantaneiro Menezes, 1992 (Characidae: Acestrorhynchinae) do Pantanal de Poconé, Mato Grosso, Brasil. Bioscience. 2010;26: 287–295.

21. Arroyave J, Stiassny MLJ. DNA barcoding reveals novel insights into pterygophagy and prey selection in distichodontid fishes (Characiformes: Distichodontidae). Ecol Evol. 2014;4: 4534–4542.

22. Winemiller KO. Spatial and temporal variation in tropical fish trophic networks. Ecol Monogr. 1990;60: 331–367.

23. Luz-Agostinho KDG, Agostinho AA, Gomes LC, Júlio-Jr HF. Influence of flood pulses on diet composition and trophic relationships among piscivorous fish in the upper Parana River floodplain. Hydrobiologia. 2008;607: 187–198. doi:10.1007/s10750-008-9390-4

24. Rossi LM. Alimentación de larvas de *Salminus maxillosus* (Val. 1840)(Pisces, Characidae). Iheringia. 1989;69: 49–59.

25. Rudstam LG, Brooking TE, Krueger SD, Jackson JR, Wetherbee L. Analysis of compensatory responses in land-locked alewives to walleye predation: a tale of two lakes. Trans Am Fish Soc. 2011;140: 1587–1603.

26. Martin JD. Atlantic salmon and alewife passage at the fishway on the Magaguadavic River, New Brunswick, during 1984. Can Manuscr Rep Fish Aquat Sci. 1987;1938: iii-7.

27. Rhodes RJ, Webb DA, McComish TS. Cannibalism by the adult Alewife (*Alosa pseudogarengus*) in southern Lake Michigan. Proceedings, Seventeenth conference on Great Lakes research, Part 1. 1974.

28. Ridgway MS, Hurley DA, Scott KA. Effects of winter temperature and predation on the abundance of alewife (*Alosa pseudoharengus*) in the Bay of Quinte, Lake Ontario. J Great Lakes Res. 1990;16: 11–20.

29. Harris JE, McBride RS. American shad feeding on spawning grounds in the St. Johns River, Florida. Trans Am Fish Soc. 2009;138: 888–898.

30. Prokopchuk I, Sentyabov E. Diets of herring, mackerel, and blue whiting in the Norwegian Sea in relation to *Calanus finmarchicus* . ICES J Mar Sci. 2006;63: 117–127.

31. Skaret G, Axelsen BE, Nøttestad L, Fernö A, Johannessen A. Herring as

cannibals. J Fish Biol. 2002;61: 1050–1052.

32. Schnack D. Causes of major changes in marine fish stocks in the Baltic Sea. Meer und Museum. 2003;17: 96–103.

33. Friedlander AM, Beets JP. Fisheries and life history characteristics of dwarf herring (*Jenkinsia lamprotaenia*) in the US Virgin Islands. Fish Res. 1997;31: 61–72.

34. De Iongh HH, Spliethoff PC, Frank VG. Feeding habits of the clupeid *Limnothrissa miodon* (Boulenger), in Lake Kivu. Hydrobiologia. 1983;102: 113–122.

35. Isumbisho M, Kaningini M, Descy JP, Baras E. Seasonal and diel variations in diet of the young stages of the fish *Limnothrissa miodon* in Lake Kivu, Eastern Africa. J Trop Ecol. 2004;20: 73–83.

36. Costalago D, Garrido S, Palomera I. Comparison of the feeding apparatus and diet of European sardines *Sardina pilchardus* of Atlantic and Mediterranean waters: ecological implications. J Fish Biol. 2005;1986: 1348–1362. doi:10.1111/jfb.12645

37. Garrido S, Ben-Hamadou R, Oliveira PB, Cunha ME, Chícharo MA, van der Lingen CD. Diet and feeding intensity of sardine *Sardina pilchardus*: correlation with satellite-derived chlorophyll data. Mar Ecol Prog Ser. 2008;354: 245–256.

38. Smith PE, Santander H, Alheit J. Comparison of the mortality rates of Pacific sardine, *Sardinops sagax*, and Peruvian anchovy, *Engraulis ringens*, eggs off Peru. Fish Bull. 1989;87: 497–508.

39. Alheit J. Egg cannibalism versus egg predation: their significance in anchovies. South African J Mar Sci. 1987;5: 467–470.

40. Hammann MG, Nevárez-Martínez MO, Green-Ruíz Y. Spawning habitat of the Pacific sardine (*Sardinops sagax*) in the Gulf of California: Egg and larval distribution 1956-1957 and 1971-1991. California Cooperative Oceanic Fisheries Investigations Report. 1998.

41. Casini M, Cardinale M, Hjelm J. Inter-annual variation in herring, *Clupea*

harengus, and sprat, *Sprattus sprattus*, condition in the central Baltic Sea: what gives the tune? Oikos. 2006;112: 638–650.

42. Köster FW, Möllmann C. Egg cannibalism in Baltic sprat *Sprattus sprattus*. Mar Ecol Prog Ser. 2000;196: 269–277.

43. Milton DA, Rawlinson NJ, Blaber SJ. Recruitment patterns and factors affecting recruitment of five species of short-lived clupeoids in the tropical South Pacific. Fish Res. 1996;26: 239–255.

44. Pájaro M, Curelovich J, Macchi GJ. Egg cannibalism in the northern population of the Argentine anchovy, *Engraulis anchoita* (Clupeidae). Fish Res. 2007;83: 253–262.

45. Pajaro M, Sanchez RP, Aubone A. Embryonic mortality due to cannibalism in the Argentine anchovy *Engraulis anchoita* Hubbs and Marini, 1935. Bol Inst Esp Oceanogr. 1998;14: 81–98.

46. Szeinfeld E V. Cannibalism and intraguild predation in clupeoids. Mar Ecol Prog Ser. 1991;79: 17–26.

47. Valdés ES, Shelton PA, Armstrong MJ, Field JG. Cannibalism in South African anchovy: egg mortality and egg consumption rates. South African J Mar Sci. 1987;5: 613–622. doi:10.2989/025776187784522595

48. Hutchings L, Barange M, Bloomer SF, Boyd AJ, Crawford RJM, Huggett JA, et al. Multiple factors affecting South African anchovy recruitment in the spawning, transport and nursery areas. South African J Mar Sci. 1998;19: 211–225.

49. Szeinfeld E V. The energetics and evolution of intraspecific predation (egg cannibalism) in the anchovy *Engraulis capensis*. Mar Biol. 1993;115: 301–308.

50. Gennotte V, Torre M, Kallianiotis A. Cannibalism in anchovy (*Engraulis encrasicolus*) in the North Aegean Sea (Greece). Rapp Comm int Mer Médit. 2007;38: 484.

51. Bachiller E, Cotano U, Ibaibarriaga L, Santos M, Irigoien X. Intraguild predation between small pelagic fish in the Bay of Biscay: impact on anchovy (*Engraulis encrasicolus* L.) egg mortality. Mar Biol. 2015;162: 1351–1369.

52. Kono N, Zenitani H. Fluctuations of abundance and survival rate during the egg and larval stages of Japanese anchovy *Engraulis japonicus* in the Seto Inland Sea (1980–2007). Fish Sci. 2012;78: 753–760.

53. Takasuka A, Oozeki Y, Kimura R, Kubota H, Aoki I. Growth-selective predation hypothesis revisited for larval anchovy in offshore waters: cannibalism by juveniles versus predation by skipjack tunas. Mar Ecol Prog Ser. 2004;278: 297–302.

54. Santander H. Relationship between anchoveta egg standing stock and parent biomass off Peru, 4–14 S. The Peruvian Anchoveta and its Upwelling Ecosystems: Three Decades of Change. ICLARM Stud Rev. 1987; 179–207.

55. Pauly D, Soriano M. Production and mortality of Anchoveta (*Engraulis ringens*) eggs of Peru. ICLARM Contrib. 1989;

56. Krautz M, Castro LR, González M. Interaction of two key pelagic species in the Humboldt Current: euphausiid predation on anchoveta eggs estimated by immunoassays. Mar Ecol Prog Ser. 2007;335: 175–185.

57. Espinoza P, Bertrand A. Revisiting Peruvian anchovy (*Engraulis ringens*) trophodynamics provides a new vision of the Humboldt Current system. Prog Oceanogr. 2008;79: 215–227.

58. Brownell CL. Cannibalistic interactions among young anchovy: a first attempt to apply laboratory behavioural observations to the field. South African J Mar Sci. 1987;5: 503–511.

59. Folkvord A, Hunter JR. Size-specific vulnerability of northern anchovy, *Engraulis mordax*, larvae to predation by fishes. Fish Bull. 1986;84: 859–869.

60. Peterman RM, Bradford MJ. Wind speed and mortality rate of a marine fish, the northern anchovy (*Engraulis mordax*). Science (80-). 1987;235: 354–356.

61. Cooke SJ, Bunt CM. Spawning and reproductive biology of the greater redhorse, *Moxostoma valenciennesi*, in the Grand River, Ontario. Can Field-Naturalist. 1999;113: 497–502.

62. Donnelly BG, Marshall BE. The biology of *Barbus mattozi* Guimaraes

(Teleostei, Cyprinidae) in a Zimbabwean reservoir. 3. Numbers, biomass and mortality. African J Aquat Sci. 2004;29: 103–106.

63. Tonn WM, Holopainen IJ, Paszkowski CA. Density-dependent effects and the regulation of crucian carp populations in single-species ponds. Ecology. 1994;75: 824–834. doi:10.2307/1941738

64. Katano O. Cannibalism on eggs by dark chub, *Zacco temmincki* (Temminck & Schlegel)(Cyprinidae). J Fish Biol. 1992;41: 655–661.

65. Katano O, Maekawa K. Individual differences in egg cannibalism in female dark chub (Pisces: Cyprinidae). Behaviour. 1996;132: 237–352. doi:10.1017/CBO9781107415324.004

66. Katano O. Spawning tactics of paired males of the dark chub, *Zacco temmincki*, reflect potential fitness costs of satellites. Environ Biol Fishes. 1992;35: 343–350.

67. Gard MF. Ontogenetic microhabitat shifts in Sacramento pikeminnow, *Ptychocheilus grandis*: reducing intraspecific predation. Aquat Ecol. 2005;39: 229–235.

68. Nakamoto RJ, Harvey BC. Spatial, seasonal, and size-dependent variation in the diet of Sacramento pikeminnow in the Eel River, northwestern California. Calif Fish Game. 2003;89: 30–45.

69. Quist MC, Bower MR, Hubert WA. Summer food habits and trophic overlap of roundtail chub and creek chub in Muddy Creek, Wyoming. Southwest Nat. 2006;51: 22–27.

70. Sousa-Santos C, Robalo J, Almada VC. Spawning behaviour of a threatened Iberian cyprinid and its implications for conservation. Acta Ethol. 2014;17: 99–106.

71. Yoshihiro B, Yoshikazu N. Distribution and dynamics of eggs and larvae in the spawning redds of pale chub, *Zacco platypus*. Japanese J Ichthyol. 2005;52: 125–132.

72. Hammer C. Feeding behaviour of roach (*Rutilus rutilus*) larvae and the fry of

perch (*Perca fluviatilis*) in Lake Lankau. Arch für Hydrobiol. 1985;103: 61–74.

73. Able KW, Hagan SM, Kovitvongsa K, Brown SA, Lamonaca JC. Piscivory by the mummichog (*Fundulus heteroclitus*): evidence from the laboratory and salt marshes. J Exp Mar Bio Ecol. 2007;345: 26–37.

74. Öztürk S. Some biological properties in the Akgöl (Fethiye-Muğla) population of the Mosquitofish *Gambusia affinis* (Baird & Girard, 1853). Turkish J Vet Anim Sci. 2003;27: 911–915.

75. Specziár A. Life history pattern and feeding ecology of the introduced eastern mosquitofish, *Gambusia holbrooki*, in a thermal spa under temperate climate, of Lake Heviz, Hungary. Hydrobiologia. 2004;522: 249–260. doi:10.1023/B:HYDR.0000029978.46013.d1

76. Nesbit DH, Meffe GK. Cannibalism frequencies in wild populations of the eastern mosquitofish (*Gambusia holbrooki*: Poeciliidae) in South Carolina. Copeia. 1993;1993: 867–870. doi:10.2307/1447254

77. Ivantsoff W. Detection of predation on Australian native fishes by *Gambusia holbrooki*. Mar Freshw Res. 1999;50: 467–468. doi:10.1071/MF98106

78. Kipling C, Frost WE. A study of the mortality, population numbers, year class strengths, production and food consumption of pike, *Esox lucius* L., in Windermere from 1944 to 1962. J Anim Ecol. 1970; 115–157.

79. Kipling C. A study of perch (*Perca fluviatilis* L.) and pike (*Esox lucius* L.) in Windermere from 1941 to 1982. J du Cons. 1984;41: 259–267.

80. Hawkins LA, Armstrong JD, Magurran AE. Aggregation in juvenile pike (*Esox lucius*): interactions between habitat and density in early winter. Ecology. 2005;19: 794–799.

81. Nilsson PA. Avoid your neighbours: size-determined spatial distribution patterns among northern pike individuals. Oikos. 2006;113: 251–258.

82. Kipling C. Changes in the population of pike (*Esox lucius*) in Windermere from 1944 to 1981. J Anim Ecol. 1983;52: 989–999.

83. Haugen TO, Winfield IJ, Vøllestad LA, Fletcher JM, James JB, Stenseth NC. Density dependence and density independence in the demography and dispersal of pike over four decades. Ecol Monogr. 2007;77: 483–502.

84. Morrow Jr J V., Miller GL, Killgore KJ. Density, size, and foods of larval northern pike in natural and artificial wetlands. North Am J Fish Manag. 1997;17: 210–214.

85. Venturelli PA, Tonn WM. Diet and growth of northern pike in the absence of prey fishes: initial consequences for persisting in disturbance-prone lakes. Trans Am Fish Soc. 2006;135: 1512–1522. doi:10.1577/T05-228.1

86. Alp A, Yeğen V, Apaydin Yağci M, Uysal R, Biçen E, Yağci A. Diet composition and prey selection of the pike, *Esox lucius*, in Civril Lake, Turkey. J Appl Ichthyol. 2008;24: 670–677.

87. Eklöv P. Effects of habitat complexity and prey abundance on the spatial and temporal distributions of perch (*Perca fluviatilis*) and pike (*Esox lucius*). Can J Fish Aquat Sci. 1997;53: 1520–1531. doi:10.1139/cjfas-54-7-1520

88. Kangur P. Food of pike, *Esox lucius* L., in Lake Peipsi. Proc Est Acad Sci Biol Ecol. 2000;49: 109–120.

89. Vronskii BB. Food of some predaceous fishes of the Amur basin (Translation). From Ref Zh Biol. 1964;

90. Beaudoin CP, Tonn WM, Prepas EE, Wassenaar LI. Individual specialization and trophic adaptability of northern pike (*Esox lucius*): an isotope and dietary analysis. Oecologia. 1999;120: 386–396.

91. Mills CA, Hurley MA. Long-term studies on the Windermere populations of perch (*Perca fluviatilis*), pike (*Esox lucius*) and Arctic charr (*Salvelinus alpinus*). Freshw Biol. 1990;23: 119–136.

92. Cucherousset J, Paillisson JM, Roussel JM. Natal departure timing from spatially varying environments is dependent of individual ontogenetic status. Naturwissenschaften. 2013;100: 761–768.

93. Banks JW. Observations on the fish population of Rostherne Mere, Cheshire. F

Stud. 1970; 357–379.

94. Grønkjær P, Skov C, Berg S. Otolith-based analysis of survival and size-selective mortality of stocked 0+ year pike related to time of stocking. J Fish Biol. 2004;64: 1625–1637.

95. Kekäläinen J, Niva T, Huuskonen H. Pike predation on hatchery-reared Atlantic salmon smolts in a northern Baltic river. Ecol Freshw Fish. 2008;17: 100–109.

96. Sharma CM, Borgstrøm R, Huitfeldt JS, Rosseland BO. Selective exploitation of large pike *Esox lucius*—effects on mercury concentrations in fish populations. Sci Total Environ. 2008;399: 33–40.

97. Wahlström E, Persson L, Diehl S, Byström P. Size-dependent foraging efficiency, cannibalism and zooplankton community structure. Oecologia. 2000;123: 138–148.

98. Cucherousset J, Paillisson JM, Cuzol A, Roussel JM. Spatial behaviour of young-of-the-year northern pike (*Esox lucius* L.) in a temporarily flooded nursery area. Ecol Freshw Fish. 2009;18: 314–322.

99. Persson L, Bertolo A, de Roos AM. Temporal stability in size distributions and growth rates of three *Esox lucius* L. populations. A result of cannibalism? J Fish Biol. 2006;69: 461–472.

100. Mann RHK. The annual food consumption and prey preferences of pike (*Esox lucius*). J Anim Ecol. 1982;51: 81–95.

101. Grimm MP. The composition of northern pike (*Esox lucius* L.) populations in four shallow waters in the Netherlands, with special reference to factors influencing 0+ pike biomass. Aquac Res. 1981;12: 61–76.

102. Owen R, Bowers E. The population dynamics of pike, *Esox lucius*, and perch, *Perca fluviatilis*, in a simple predator-prey system. Environ Biol Fishes. 1992;34: 65–78.

103. Edeline E, Carlson SM, Stige LC, Winfield IJ, Fletcher JM, James JB, et al. Trait changes in a harvested population are driven by a dynamic tug-of-war between natural and harvest selection. Proc Natl Acad Sci. 2007;104: 15799–15804.

104. Soupir CA, Brown ML, Kallemeyn LW. Trophic ecology of largemouth bass and northern pike in allopatric and sympatric assemblages in northern boreal lakes. Can J Zool. 2000;78: 1759–1766.

105. Pedreschi D, Mariani S, Coughlan J, Voigt CC, O'Grady M, Caffrey J, et al. Trophic flexibility and opportunism in pike *Esox lucius*. J Fish Biol. 2015;87: 876–894.

106. Nilsson J-Å, Engstedt O, Larsson P. Wetlands for northern pike (*Esox lucius* L.) recruitment in the Baltic Sea. Hydrobiologia. 2014;721: 145–154.

107. Panek FM, Weis JS. Diet of the Eastern Mudminnow (*Umbra pygmaea* DeKay) from two geographically distinct populations within the North American native range. Northeast Nat. 2013;20: 37–48.

108. Jurado-Molina J, Livingston PA, Galluci VF. Sensitivity analysis of the multispecies virtual population analysis model parameterized for a system of trophically-linked species from the eastern Bering Sea. Ciencias Mar. 2004;30: 285–296.

109. Daan N. A quantitative analysis of the food intake of North Sea cod, *Gadus morhua*. Netherlands J Sea Res. 1973;6: 479–517.

110. Blom G, Folkvord A. A snapshot of cannibalism in 0-group Atlantic cod (*Gadus morhua*) in a marine pond. J Appl Ichthyol. 1997;13: 177–181.

111. Fromentin JM, Gjøsæter J, Bjørnstad ON, Stenseth NC. Biological processes and environmental factors regulating the dynamics of the Norwegian Skagerrak cod populations since 1919. ICES J Mar Sci. 2000;57: 330–338.

112. Patriquin DG. Biology of *Gadus morhua* in Ogac Lake, a landlocked fiord on Baffin Island. J Fish Board Canada. 1967;24: 2573–2594.

113. Bogstad B, Lilly GR, Mehl S, Palsson OK, Stefánsson G. Cannibalism and year-class strength in Atlantic cod (*Gadus morhua* L.). In: Akobsson J, Atthorsson OS, Beverton RJH, editors. Arcto-boreal ecosystems (Barents Sea, Iceland, and eastern Newfoundland). 1994. pp. 576–599.

114. Nakken O. Causes of trends and fluctuations in the Arcto-Norwegian stock.

Symposium on Cod and Climate Change. 1994. pp. 212–228.

115. Uzars D, Plikshs M. Cod (*Gadus morhua* L.) cannibalism in the Central Baltic: interannual variability and influence of recruit abundance and distribution. ICES J Mar Sci. 2000;57: 324–329.

116. Bromley PJ, Watson T, Hislop JRG. Diel feeding patterns and the development of food webs in pelagic 0-group cod (*Gadus morhua* L.), haddock (*Melanogrammus aeglefinus* L.), whiting (*Merlangius merlangus* L.), saithe (*Pollachius virens* L.), and Norway pout (Trisopte. ICES J Mar Sci. 1997;54: 846–853.

117. Grant SM, Brown JA. Diel foraging cycles and interactions among juvenile Atlantic cod (*Gadus morhua*) at a nearshore site in Newfoundland. Can J Fish Aquat Sci. 1998;55: 1307–1316.

118. Knickle DC, Rose GA. Dietary niche partitioning in sympatric gadid species in coastal Newfoundland: evidence from stomachs and CN isotopes. Environ Biol Fishes. 2014;97: 343–355.

119. Anderson JT, Gregory RS. Factors regulating survival of northern cod (NAFO 2J3KL) during their first 3 years of life. ICES J Mar Sci. 2000;57: 349–359.

120. Vazquez FJ, Paz FJ, Casas JM, Cardenas E de, Alvarez E, Arroyo AF. Feed of american plaice greenland halibut redfish and cod in flemish cap north atlantic ocean in july 1988. Bol Instituro Esp Oceanogr. 1989;5: 43–58.

121. Magnussen E. Food and feeding habits of cod (*Gadus morhua*) on the Faroe Bank. ICES J Mar Sci. 2011;68: 1909–1917.

122. Pachur ME, Horbowy J. Food composition and prey selection of cod, *Gadus morhua* (Actinopterygii: gadiformes: gadidae), in the southern Baltic Sea. Acta Ichtyologica Piscat. 2013;43: 109–118.

123. Almqvist G, Strandmark AK, Appelberg M. Has the invasive round goby caused new links in Baltic food webs? Environ Biol Fishes. 2010;89: 79–93.

124. Castonguay M, Chabot C, Fréchet A, Hammill MO, Morissette L. Main prey and predators and estimates of mortality of Atlantic cod (*Gadus morhua*) in the

northern Gulf of St. Lawrence during the mid-1980s, mid-1990s, and early 2000s. Fish Ocean Canada, Sci. 2006;

125. Øiestad V, Kvenseth PG, Folkvord A. Mass production of Atlantic cod juveniles *Gadus morhua* in a Norwegian saltwater pond. Trans Am Fish Soc. 1985;114: 590–595.

126. Skreslet S, Albrigtsen I, Andersen AP, Kolbeinshavn A, Pedersen T, Unstad K. Migration, growth and survival in stocked and wild cod (*Gadus morhua* L.). The Vestfjord region, North Norway Stock Enhancement and Sea Ranching. Oxford: Fishing News Books; 1999. pp. 306–314.

127. Lindstrøm U, Smout S, Howell D, Bogstad B. Modelling multi-species interactions in the Barents Sea ecosystem with special emphasis on minke whales and their interactions with cod, herring and capelin. Deep Sea Res Part II. 2009;56: 2068–2079.

128. Hanson JM. Ontogenetic diet comparison of Atlantic Cod and White Hake occurring at historically low population abundances. Trans Am Fish Soc. 2011;140: 1070–1077.

129. Salvanes AG V. Pollack (*Pollachius pollachius*) stock size development and potential influence on cod (*Gadus morhua*) mariculture in a west Norwegian fjord. Fish Res. 1995;24: 223–242.

130. Linehan JE, Gregory RS, Schneider DC. Predation risk of age-0 cod (*Gadus*) relative to depth and substrate in coastal waters. J Exp Mar Bio Ecol. 2001;263: 25–44.

131. Tsou TS, Collie JS. Predation-mediated recruitment in the Georges Bank fish community. ICES J Mar Sci. 2001;58: 994–1001.

132. Støttrup JG, Overton JL, Paulsen H, Möllmann C, Tomkiewicz J, Pedersen PB, et al. Rationale for restocking the eastern Baltic cod stock. Rev Fish Sci. 2008;16: 68–64.

133. Casas JM, Paz P. Recent changes in the feeding of cod (*Gadus morhua*) off the Flemish Cap, Newfoundland 1989–1993. ICES J Mar Sci. 1996;53: 750–756.

134. Fromentin JM, Stenseth NC, Gjøsæter J, Bjørnstad ON, Falck W, Johannessen T. Spatial patterns of the temporal dynamics of three gadoid species along the Norwegian Skagerrak coast. Mar Ecol Prog Ser. 1997;155: 209–222.

135. Jarre A, Wieland K, MacKenzie B, Hinrichsen HH, Plikshs M, Aro E. Stock recruitment relationships for cod (*Gadus morhua* L.) in the central Baltic Sea incorporating environmental variability. Arch Fish Mar Res. 2000;48: 97–123.

136. Kjesbu OS, Bogstad B, Devine JA, Gjøsæter H, Howell D, Ingvaldsen RB, et al. Synergies between climate and management for Atlantic cod fisheries at high latitudes. Proc Natl Acad Sci. 2014;111: 3478–3483.

137. Hardie DC, Hutchings JA. The ecology of Atlantic cod (*Gadus morhua*) in Canadian Arctic lakes. Arctic. 2011;64: 137–150.

138. Kanapathippillai P, Berg E, dos Santos J, Gulliksen B, Pedersen T. The food consumption of cod, *Gadus morhua* L., in a high-latitude enhancement area. Aquac Res. 1994;25: 65–76.

139. Mehl S. The Northeast Arctic cod stock's place in the Barents Sea ecosystem in the 1980s: an overview. Polar Res. 1991;10: 525–534.

140. Hjermann DØ, Stenseth NC, Ottersen G. The population dynamics of Northeast Arctic cod (*Gadus morhua*) through two decades: an analysis based on survey data. Can J Fish Aquat Sci. 2004;61: 1747–1755.

141. Steingrund P, Mouritsen R, Reinert J, Gaard E, Hátún H. Total stock size and cannibalism regulate recruitment in cod (*Gadus morhua*) on the Faroe Plateau. ICES J Mar Sci. 2010;67: 111–124.

142. Link JS, Garrison LP. Trophic ecology of Atlantic cod *Gadus morhua* on the northeast US continental shelf. Mar Ecol Prog Ser. 2002;227: 109–123.

143. Neuenfeldt S, Köster FW. Trophodynamic control on recruitment success in Baltic cod: the influence of cannibalism. ICES J Mar Sci. 2000;57: 300–309.

144. Yaragina NA, Bogstad B, Kovalev YA. Variability in cannibalism in Northeast Arctic cod (*Gadus morhua*) during the period 1947–2006. Mar Biol Res. 2009;5: 75–85.

145. Link JS, Lucey SM, Melgey JH. Examining cannibalism in relation to recruitment of silver hake *Merluccius bilinearis* in the US northwest Atlantic. Fish Res. 2012;114: 31–41.

146. Rindorf A. Diel feeding pattern of whiting in the North Sea. Mar Ecol Prog Ser. 2003;249: 265–276.

147. Christensen V. A model of throphic interactions in the North Sea in 1981, the year of the stomach. Dana. 1995;11: 1–28.

148. Cabral HN, Murta AG. The diet of blue whiting, hake, horse mackerel and mackerel off Portugal. J Appl Ichthyol. 2002;18: 14–23.

149. Urban D. Food habits of Pacific cod and walleye pollock in the northern Gulf of Alaska. Mar Ecol Prog Ser. 2012;469: 215–222.

150. Livingston PA. Importance of predation by groundfish, marine mammals and birds on walleye pollock *Theragra chalcogramma* and Pacific herring *Clupea pallasi* in the eastern Bering Sea. Mar Ecol Prog Ser. 1993;102: 205–215.

151. Ciannelli L, Brodeur RD, Swartzman GL, Salo S. Physical and biological factors influencing the spatial distribution of age-0 walleye pollock (*Theragra chalcogramma*) around the Pribilof Islands, Bering Sea. Deep Sea Res Part II. 2002;49: 6109–6126.

152. Yamamura O, Yabuki K, Shida O, Watanabe K, Honda S. Spring cannibalism on 1 year walleye pollock in the Doto area, northern Japan: is it density dependent? J Fish Biol. 2001;59: 645–656.

153. Yamamura O, Funamoto T, Chimura M, Honda S, Oshima T. Interannual variation in diets of walleye pollock in the Doto area, in relation to climate variation. Mar Ecol Prog Ser. 2013;491: 221–234.

154. Yamamura O, Honda S, Shida O, Hamatsu T. Diets of walleye pollock *Theragra chalcogramma* in the Doto area, northern Japan: ontogenetic and seasonal variations. Mar Ecol Prog Ser. 2002;238: 187–198.

155. Shida O, Miyake H, Kaneta T, Ishida R, Miyashita K. Winter distribution of young walleye pollock *Theragra chalcogramma* investigated with quantitative

echosounder on the Pacific coast of eastern Hokkaido, Japan. Nippon Suisan Gakkaishi. 2008;74: 152–160.

156. Duffy-Anderson JT, Ciannelli L, Honkalehto T, Bailey KM, Sogard SM, Springer AM, et al. Distribution of age-1 and age-2 walleye pollock in the Gulf of Alaska and eastern Bering Sea: sources of variation and implications for higher trophic levels. The Big Fish Bang: Proceedings of the 26th Annual Larval Fish Conference Institute of Marine Research, Bergen, Norway. 2003. pp. 381–394.

157. De Robertis A, Cokelet ED. Distribution of fish and macrozooplankton in ice-covered and open-water areas of the eastern Bering Sea. Deep Sea Res Part II. 2012;65: 217–229.

158. Winter A, Swartzman G, Ciannelli L. Early-to late-summer population growth and prey consumption by age-0 pollock (*Theragra chalcogramma*), in two years of contrasting pollock abundance near the Pribilof Islands, Bering Sea. Fish Oceanogr. 2005;14: 307–320.

159. Mueter FJ, Bond NA, Ianelli JN, Hollowed AB. Expected declines in recruitment of walleye pollock (*Theragra chalcogramma*) in the eastern Bering Sea under future climate change. ICES J Mar Sci. 2011;68: 1284–1296.

160. Boldt JL, Buckley TW, Rooper CN, Aydin K. Factors influencing cannibalism and abundance of walleye pollock (*Theragra chalcogramma*) on the eastern Bering Sea shelf, 1982–20. Fish Bull. 2012;110: 293–306.

161. Dwyer DA, Bailey KM, Livingston PA. Feeding habits and daily ration of walleye pollock (*Theragra chalcogramma*) in the eastern Bering Sea, with special reference to cannibalism. Can J Fish Aquat Sci. 1987;44: 1972–1984.

162. Bailey KM. Interaction between the vertical distribution of juvenile walleye pollock *Theragra chalcogramma* in the eastern Bering Sea, and cannibalism. Mar Ecol Prog Ser. 1989;53: 205–213.

163. Livingston PA. Key fish species, northern fur seals, *Callorhinus ursinus*, and fisheries interactions involving walleye pollock, *Theragra chalcogramma*, in the eastern Bering Sea. J Fish Biol. 1989;35: 179–186.

164. Mueter FJ, Ladd C, Palmer MC, Norcross BL. Bottom-up and top-down controls of walleye pollock (*Theragra chalcogramma*) on the Eastern Bering Sea shelf. Prog Oceanogr. 2006;68: 152–183.

165. Hunsicker ME, Ciannelli L, Bailey KM, Zador S, Stige LC. Climate and demography dictate the strength of predator-prey overlap in a subarctic marine ecosystem. PLoS One. 2013;8: e66025.

166. Hunt GL, Coyle KO, Eisner LB, Farley E V., Heintz RA, Mueter F, et al. Climate impacts on eastern Bering Sea foodwebs: a synthesis of new data and an assessment of the Oscillating Control Hypothesis. ICES J Mar Sci. 2011;68: 1230–1243.

167. Livingston PA, Jurado-Molina J. A multispecies virtual population analysis of the eastern Bering Sea. ICES J Mar Sci. 2000;57: 294–299.

168. Kim S, Gunderson DR. Cohort dynamics of walleye pollock in Shelikof Strait, Gulf of Alaska, during the egg and larval periods. Trans Am Fish Soc. 1989;118: 264–273.

169. White VC, Morado JF, Friedman CS. Ichthyophonus-infected walleye pollock *Theragra chalcogramma* (Pallas) in the eastern Bering Sea: a potential reservoir of infections in the North Pacific. J Fish Dis. 2014;37: 641–655.

170. Wespestrad VG, Fritz LW, Ingraham WJ, Megrey BA. On relationships between cannibalism, climate variability, physical transport, and recruitment success of Bering Sea walleye pollock (*Theragra chalcogramma*). ICES J Mar Sci. 2000;57: 272–278.

171. Brodeur RD, Bailey KM, Yoshiro Y, Yamashita Y, Oozeki Y. Predation on the early life stages of marine fish: A case study on walleye pollock in the Gulf of Alaska. Survival Strategies in Early Life Stages of Marine Resources, Yokohama Japon. 1994.

172. Brodeur RD, Merati N. Predation on walleye pollock (*Theragra chalcogramma*) eggs in the western Gulf of Alaska: the roles of vertebrate and invertebrate predators. Mar Biol. 1993;117: 483–493. doi:10.1007/BF00349324

173. Schabetsberger R, Brodeur RD, Honkalehto T, Mier KL. Sex-biased egg cannibalism in spawning walleye pollock: the role of reproductive behavior. Environ Biol Fishes. 1999;54: 175–190.

174. Brodeur RD, Wilson MT, Ciannelli L. Spatial and temporal variability in feeding and condition of age-0 walleye pollock (*Theragra chalcogramma*) in frontal regions of the Bering Sea. ICES J Mar Sci. 2000;57: 256–264.

175. Lang GM, Brodeur RD, Napp JM, Schabetsberger R. Variation in groundfish predation on juvenile walleye pollock relative to hydrographic structure near the Pribilof Islands, Alaska. ICES J Mar Sci. 2000;57: 265–271.

176. Brodeur RD, Picquelle SJ, Blood DM, Merati N. Walleye pollock egg distribution and mortality in the western Gulf of Alaska. Fish Oceanogr. 1996;5: 92–111.

177. Fuita T, Kitagawa D, Okuyama Y, Ishito Y, Inada T, Jin Y. Diets of the demersal fishes on the shelf off Iwate, northern Japan. Mar Biol. 1995;123: 219–233.

178. Harrison PM, Gutowsky LFG, Martins EG, Patterson DA, Leake A, Cooke SJ, et al. Diel vertical migration of adult burbot: a dynamic trade-off among feeding opportunity, predation avoidance, and bioenergetic gain. Can J Fish Aquat Sci. 2013;70: 1765–1774.

179. Gallagher CP, Dick TA. Winter feeding ecology and the importance of cannibalism in juvenile and adult burbot (*Lota lota*) from the Mackenzie Delta, Canada. Hydrobiologia. 2015;757: 73–88.

180. Jacobs GR, Madenjian CP, Bunnell DB, Holuszko JD. Diet of lake trout and burbot in northern Lake Michigan during spring: evidence of ecological interaction. J Great Lakes Res. 2010;36: 312–317.

181. Blaber SJM, Bulman CM. Diets of fishes of the upper continental slope of eastern Tasmania: content, calorific values, dietary overlap and trophic relationships. Mar Biol. 1987;95: 345–356.

182. Bulman CM, Blaber SJM. Feeding ecology of *Macruronus novaezelandiae* (Hector)(Teleostei: Merlucciidae) in south-eastern Australia. Mar Freshw Res.

1986;37: 621–639.

183. Garrison LP, Link JS. Diets of five hake species in the northeast United States continental shelf ecosystem. Mar Ecol Prog Ser. 2000;204: 243–255.

184. Koeller PA, Coates-Markle L, Neilson JD. Feeding ecology of juvenile (age-0) silver hake (*Merluccius bilinearis*) on the Scotian Shelf. Can J Fish Aquat Sci. 1989;46: 1762–1768. doi:10.1139/f89-223

185. Link JS, Garrison LP. Changes in piscivory associated with fishing induced changes to the finfish community on Georges Bank. Fish Res. 2002;55: 71–86.

186. Macpherson E, Gordoa A. Effect of prey densities on cannibalism in Cape hake (*Merluccius capensis*) off Namibia. Mar Biol. 1994;119: 145–149. doi:10.1007/BF00350116

187. Pillar SC, Barange M. Feeding selectivity of juvenile Cape hake *Merluccius capensis* in the southern Benguela. South African J Mar Sci. 1993;13: 255–268.

188. Pillar SC, Wilkinson IS. The diet of Cape hake *Merluccius capensis* on the south coast of South Africa. South African J Mar Sci. 1995;15: 225–239.

189. Neira S, Arancibia H, Cubillos LA. Comparative analysis of trophic structure of commercial fishery species off Central Chile in 1992 and 1998. Ecol Modell. 2004;172: 233–248.

190. Payá I, Ehrhardt NM. Comparative sustainability mechanisms of two hake (*Merluccius gayi gayi* and *Merluccius australis*) populations subjected to exploitation in Chile. Bull Mar Sci. 2005;76: 261–286.

191. Guevara-Carrasco R, Lleonart J. Dynamics and fishery of the Peruvian hake: Between nature and man. J Mar Syst. 2008;71: 249–259.

192. Orrego H, Mendo J. Variación interanual de la dieta de la merluza *Merluccius gayi* peruanus (Guitchenot) en la costa peruana. Ecol Apl. 2012;11: 103–116.

193. Cubillos LA, Alarcón C, Arancibia H. Selectividad por tamaño de las presas en merluza común (*Merluccius gayi* gayi), zona centro-sur de Chile (1992-1997). Investig Mar. 2007;35: 55–69.

194. San Martín MA, Cubillos LA, Saavedra JC. The spatio-temporal distribution of juvenile hake (*Merluccius gayi* gayi) off central southern Chile (1997–2006). Aquat Living Resour. 2011;24: 161–168.

195. Angelescu V, Prenski LB. Ecología trófica de la merluza común del Mar Argentino (Merlucciidae, *Merluccius hubbsi*). Parte 2. Dinámica de la alimentación analizada sobre la base de las condiciones ambientales, la estructura y las evaluaciones de los efectivos en su área de d. INIDEP-Serie Contrib. 1987;561: 205.

196. Ocampo Reinaldo M, González R, Romero MA. Feeding strategy and cannibalism of the Argentine hake *Merluccius hubbsi*. J Fish Biol. 2011;79: 1795–1814.

197. Sanchez F. ALimentacion de la Merluza (*Merluccius hubbsi*) en el Golfo de San Jorge y Aguas Adyacentes. INIDEP Inf técnico. 2009;75: 1–21.

198. Belleggia M, Figueroa DE, Irusta G, Bremec C. Spatio-temporal and ontogenetic changes in the diet of the Argentine hake *Merluccius hubbsi*. J Mar Biol Assoc United Kingdom. 2014;94: 1701–1710.

199. Cartes JE, Hidalgo M, Papiol V, Massutí E, Moranta J. Changes in the diet and feeding of the hake *Merluccius merluccius* at the shelf-break of the Balearic Islands: influence of the mesopelagic-boundary community. Deep Sea Res Part I Oceanogr Res Pap. 2009;56: 344–365.

200. Stagioni M, Montanini S, Vallisneri M. Feeding habits of European hake, *Merluccius merluccius* (Actinopterygii: Gadiformes: Merlucciidae), from the northeastern Mediterranean Sea. Acta Ichtyologica Piscat. 2011;41: 277–284.

201. Mahe K, Amara R, Bryckaert T, Kacher M, Brylinski JM. Ontogenetic and spatial variation in the diet of hake (*Merluccius merluccius*) in the Bay of Biscay and the Celtic Sea. ICES J Mar Sci. 2007;64: 1210–1219.

202. Preciado I, Punzón A, Velasco F. Spatio-temporal variability in the cannibalistic behaviour of European hake *Merluccius merluccius*: the influence of recruit abundance and prey availability. J Fish Biol. 2015;86: 1319–1334.

203. Guichet R. The diet of European hake (*Merluccius merluccius*) in the northern part of the Bay of Biscay. ICES J Mar Sci. 1995;52: 21–21.

204. Buckley TW, Livingston PA. Geographic variation in the diet of Pacific hake, with a note on cannibalism. Calif Coop Ocean Fish Investig Rep. 1997; 53–62.

205. Acuña Plavan A, Sellanes J, Rodríguez L, Burone L. Feeding ecology of *Urophycis brasiliensis* on the Uruguayan coast of the Río de la Plata estuary. J Appl Ichthyol. 2007;23: 231–239.

206. Lavin PA, McPhail JD. Adaptive divergence of trophic phenotype among freshwater populations of the threespine stickleback (*Gasterosteus aculeatus*). Can J Fish Aquat Sci. 1986;43: 2455–2463.

207. Mori S. Factors associated with and fitness effects of nest-raiding in the three-spined stickleback, *Gasterosteus aculeatus*, in a natural situation. Behaviour. 1995;132: 1022–1023.

208. Whoriskey FG, Fitzgerald GJ. Sex, cannibalism and sticklebacks. Behav Ecol Sociobiol. 1985;18: 15–18.

209. Candolin U. Changes in expression and honesty of sexual signalling over the reproductive lifetime of sticklebacks. Proc R Soc London B. 2000;267: 2425–2430.

210. Foster SA. Understanding the evolution of behavior in threespine stickleback: the value of geographic variation. Behaviour. 1995;132: 1107–1129.

211. Foster SA. Diversionary displays of paternal stickleback. Behav Ecol Sociobiol. 1988;22: 335–340.

212. Hyatt KD, Ringler NH. Role of nest raiding and egg predation in regulating population density of threespine sticklebacks (*Gasterosteus aculeatus*) in a coastal British Columbia lake. Can J Fish Aquat Sci. 1989;46: 372–383.

213. Foster SA, Baker JA. Evolutionary interplay between ecology, morphology and reproductive behavior in threespine stickleback, *Gasterosteus aculeatus*. Ecomorphology of fishes. 1995. pp. 213–223.

214. Foster SA. Inference of evolutionary pattern: diversionary displays of three-spined sticklebacks. Behav Ecol. 1994;5: 114–121.

215. Fitzgerald GJ. The role of cannibalism in the reproductive ecology of the threespine stickleback. Ethology. 1991;89: 177–194.

216. Kynard BE. Breeding behavior of a lacustrine population of threespine sticklebacks (*Gasterosteus aculeatus* L.). Behaviour. 1978;67: 178–206.

217. Foster SA, Garcia VB, Town MY. Cannibalism as the cause of an ontogenetic shift in habitat use by fry of the threespine stickleback. Oecologia. 1988;74: 577–585.

218. Ridgway MS, McPhail JD. Raiding shoal size and a distraction display in male sticklebacks (*Gasterosteus*). Can J Zool. 1988;66: 201–205.

219. Sillett KB, Foster SA. Ontogenetic niche shifts in two populations of juvenile threespine stickleback, *Gasterosteus aculeatus*, that differ in pelvic spine morphology. Oikos. 2000;91: 468–476.

220. Sparkes TC, Rush V, Foster SA. Reproductive costs, condition and carotenoid-based colour in natural populations of threespine stickleback (*Gasterosteus aculeatus*). Ecol Freshw Fish. 2008;17: 292–302.

221. Gomagano D, Kohda M. Partial filial cannibalism enhances initial body condition and size in paternal care fish with strong male-male competition. Ann Zool Fennici. 2008;45: 55–65.

222. Mlewa CM, Green JM. Biology of the marbled lungfish, *Protopterus aethiopicus* Heckel, in Lake Baringo, Kenya. Afr J Ecol. 2004;42: 338–345.

223. Payne SL, Pearson ED. Feeding preferences of postlarval longnose gar (*Lepisosteus osseus*) of the Ohio River. Trans Kentucky Acad Sci. 1981;42: 119–131.

224. Johnson AK, Richards RA, Cullen DW, Sutherland SJ. Growth, reproduction, and feeding of large monkfish, *Lophius americanus*. ICES J Mar Sci. 2008;67: 1306–1315.

225. Laurenson CH, Priede IG. The diet and trophic ecology of anglerfish *Lophius piscatorius* at the Shetland Islands, UK. J Mar Biol Assoc United Kingdom. 2005;85: 419–424.

226. Bernal A, Olivar MP, Maynou F, de Puelles MLF. Diet and feeding strategies of mesopelagic fishes in the western Mediterranean. Prog Oceanogr. 2015;135: 1–17.

227. Belk MC, Habit E, Ortiz-Sandoval JJ, Sobenes C, Combs EA. Ecology of *Galaxias platei* in a depauperate lake. Ecol Freshw Fish. 2014;23: 615–621.

228. Cadwallader PL. Distribution and ecology of the Canterbury mudfish, *Neochanna burrowsius* (Phillipps)(Salmoniformes: Galaxiidae). J R Soc New Zeal. 1975;5: 21–30.

229. Gjøsæter H, Bogstad B, Tjelmeland S. Ecosystem effects of the three capelin stock collapses in the Barents Sea. Mar Biol Res. 2009;5: 40–53.

230. Slotte A, Mikkelsen N, Gjøsæter H. Egg cannibalism in Barents Sea capelin in relation to a narrow spawning distribution. J Fish Biol. 2006;69: 187–202.

231. Dolgov A. The role of capelin (*Mallotus villosus*) in the foodweb of the Barents Sea. ICES J Mar Sci. 2002;59: 1034–1045.

232. Vinni M, Lappalainen J, Malinen T, Peltonen H. Seasonal bottlenecks in diet shifts and growth of smelt in a large eutrophic lake. J Fish Biol. 2004;64: 567–579.

233. Doherty D, McCarthy TK. he ecology and conservation of European smelt (*Osmerus eperlanus* L.) from Waterford Estuary, in southeastern Ireland. Biol Environ Proc R Irish Acad. 2004; 125–130.

234. Gorman OT. Changes in a population of exotic rainbow smelt in Lake Superior: boom to bust, 1974–2005. J Great Lakes Res. 2007;33: 75–90.

235. O’Brien TP, Taylor WW, Roseman EF, Madenjian CP, Riley SC. Ecological factors affecting rainbow smelt recruitment in the main basin of Lake Huron, 1976–2010. Trans Am Fish Soc. 2014;143: 784–795.

236. Henderson BA, Nepszy SJ. Factors affecting recruitment and mortality rates of rainbow smelt (*Osmerus mordax*) in Lake Erie, 1963–85. J Great Lakes Res. 1989;15: 357–366.

237. Stritzel Thomson JL, Parrish DL, Parker-Stetter SL, Rudstam LG, Sullivan PJ. Growth rates of rainbow smelt in Lake Champlain: effects of density and diet. Ecol Freshw Fish. 2011;20: 503–512.

238. Parker-Stetter SL, Thomson JLS, Rudstam LG, Parrish DL, Sullivan PJ. Importance and predictability of cannibalism in rainbow smelt. Trans Am Fish Soc. 2007;136: 227–237.

239. He X, LaBar GW. Interactive effects of cannibalism, recruitment, and predation on rainbow smelt in Lake Champlain: a modeling synthesis. J Great Lakes Res. 1994;20: 289–298.

240. Lantry BF, Stewart DJ. Population dynamics of rainbow smelt (*Osmerus mordax*) in Lakes Ontario and Erie: a modeling analysis of cannibalism effects. Can J Fish Aquat Sci. 2000;57: 1594–1606.

241. Link JS, Lucey SM, Melgey JH. Examining cannibalism in relation to recruitment of silver hake *Merluccius bilinearis* in the US northwest Atlantic. Fish Res. 2012;114: 31–41.

242. Eigaard OR, Deurs M V., Behrens J, Bekkevold D, Brander K, Plambech M, et al. Prey or predator–expanding the food web role of sandeel (*Ammodytes marinus*). Mar Ecol Prog Ser. 2014;516: 267–273.

243. Yamada H, Tsumoto K, Kuno M. Cannibalistic mortality of larval sand eel *Ammodytes personatus* by adults in Ise Bay central Japan. Bull Japanese Soc Sci Fish. 1998;64: 807–814.

244. Raventos N. Age, growth and reproductive parameters of the Mediterranean cardinal fish, *Apogon imberbis*. J Appl Ichthyol. 2007;23: 675–678.

245. Mazzoldi C, Randieri A, Mollica E, Rasotto MB. Notes on the reproduction of the cardinalfish *Apogon imberbis* from Lachea Island, Central Mediterranean, Sicily, Italy. Vie Milieuu. 2008;58: 63–66.

246. Kume G, Yamaguchi A, Aoki I. Reproductive biology of the paternal mouthbrooding cardinalfish *Apogon lineatus* in Tokyo Bay, Japan. Fish Sci. 2002;68: 457–458.

247. Kume G, Yamaguchi A, Taniuchi T. Feeding Habits of the Cardinalfish *Apogon lineatus* in Tokyo Bay, Japan. Fish Sci. 1999;65: 420–423.

248. Kume G, Yamaguchi A, Taniuchi T. Filial cannibalism in the paternal mouthbrooding cardinalfish *Apogon lineatus*: egg production by the female as the nutrition source for the mouthbrooding male. Environ Biol Fishes. 2000;58: 233–236.

249. Kume G, Yamaguchi A, Aoki I. Geographic variations in feeding habits of the cardinalfish *Apogon lineatus*. Bull Fac Fish Univ. 2003;84: 39–46.

250. Kume G, Yamaguchi A, Aoki I, Taniuchi T. Reproductive biology of the cardinalfish *Apogon lineatus* in Tokyo Bay, Japan. Fish Sci. 2000;66: 947–954.

251. Rueger T, Gardiner NM, Jones GP. Relationships between pair formation, site fidelity and sex in a coral reef cardinalfish. Behav Processes. 2014;107: 119–126.

252. Okuda N, Takeyama T, Yanagisawa Y. Age-specific filial cannibalism in a paternal mouthbrooding fish. Behav Ecol Sociobiol. 1997;41: 363–369.

253. Okuda N, Yanagisawa Y. Filial cannibalism by mouthbrooding males of the cardinal fish, *Apogon doederleini*, in relation to their physical condition. Environ Biol Fishes. 1996;45: 397–404. doi:10.1007/BF00002532

254. Okuda N, Yanagisawa Y. Filial cannibalism in a paternal mouthbrooding fish in relation to mate availability. Anim Behav. 1996;52: 307–314. doi:10.1006/anbe.1996.0176

255. Takeyama T, Okuda N, Yanagisawa Y. Seasonal pattern of filial cannibalism by *Apogon doederleini* mouthbrooding males. J Fish Biol. 2002;61: 633–644.

256. Okuda N, Myazaki M, Yanagisawa Y. Sexual difference in buccal morphology of the paternal mouthbrooding cardinalfish *Apogon doederleini*. Zoolog Sci. 2002;19: 801–807.

257. Okuda N, Fukumori K, Yanagisawa Y. Male ornamentation and its condition-dependence in a paternal mouthbrooding cardinalfish with extraordinary sex roles. J Ethol. 2003;21: 153–159.

258. Okuda N. Sex roles are not always reversed when the potential reproductive rate is higher in females. Am Nat. 1999;153: 540–548.

259. Okuda N. The costs of reproduction to males and females of a paternal mouthbrooding cardinalfish *Apogon notatus*. J Fish Biol. 2001;58: 776–787.

260. Kolm N, Berglund A. Sex-specific territorial behaviour in the Banggai cardinalfish, *Pterapogon kaunderni*. Environ Biol Fishes. 2004;70: 375–379.

261. Pakhomov EA. Diet of two Antarctic dragonfish (Pisces: Bathydraconidae) from the Indian sector of the Southern Ocean. Antarct Sci. 1998;10: 55–61.

262. Adams AJ, Wolfe RK. Cannibalism of juveniles by adult common snook (*Centropomus undecimalis*). Gulf Mex Sci. 2006;24: 11.

263. Barbour AB, Adams AJ, Lorenzen K. Emigration-corrected seasonal survival of a size-structured fish population in a nursery habita. Mar Ecol Prog Ser. 2014;514: 191–205.

264. Moroshi Y, Sasaki K. Intensive cannibalism and feeding on bregmacerotids in *Champsodon snyderi* (Champsodontidae): evidence for pelagic predation. Ichthyol Res. 2003;50: 387–390.

265. Kraak SBM. A quantitative description of the reproductive biology of the Mediterranean blenny *Aidablennius sphynx* (Teleostei, Blenniidae) in its natural habitat. Environ Biol Fishes. 1996;46: 329–342.

266. Vinyoles D, Sostoa A. Egg cannibalism in river blennies: the role of natural prey availability. J Fish Biol. 1999;55: 1223–1232.

267. Gasith A, Goren M. Habitat availability, reproduction and population dynamics of the fresh water blenny *Salaria fluviatilis* (Asso, 1801) in Lake Kinneret, Israel. Electron J Ichthyol. 2009;2: 34–46.

268. Fabre N, García-Galea E, Vinyoles D. Parents’ presence affects embryos’

development in *Salaria fluviatilis* (Asso, 1801), a fish with parental care. Anim Biol. 2014;64: 295–309.

269. Gonçlalves EJ, Almada VC. Sex differences in resource utilization by the peacock blenny. J Fish Biol. 1997;51: 624–633.

270. Pelham ME, Pierce CL, Larscheid JG. Diet dynamics of the juvenile piscivorous fish community in Spirit Lake, Iowa, USA, 1997–1998. Ecol Freshw Fish. 2001;10: 198.

271. Copp GH, Fox MG, Kovac V. Growth, morphology and life history traits of a cool-water European population of pumpkinseed *Lepomis gibbosus*. Arch für Hydrobiol. 2002;155: 585–614.

272. Neff BD. Decisions about parental care in response to perceived paternity. Nature. 2003;422: 716–719. doi:10.1038/nature01528

273. Warren DJ, Cogdon BD, Mueller KW. Diet analysis of smallmouth bass (*Micropterus dolomieu*) and largemouth bass (*Micropterus salmoides*) from Spencer Lake, Blakely Island, Washington. Washingt Dep Fish Wildl Tech Rep FTP. 2002; 10.

274. Clady MD. Food habits of yellow perch, smallmouth bass and largemouth bass in two unproductive lakes in northern Michigan. Am Midl Nat. 1974;91: 453–459.

275. Smith KL, Miner JG, Wiegmann DD, Newman SP. Individual differences in exploratory and antipredator behaviour in juvenile smallmouth bass (*Micropterus dolomieu*). Behaviour. 2009;146: 283–294.

276. Weyl OL, Hecht T. A successful population of largemouth bass, *Micropterus salmoides*, in a subtropical lake in Mozambique. Environ Biol Fishes. 1999;54: 53–66. doi:10.1023/A:1007452320609

277. Hickley P, North R, Muchiri SM, Harper DM. The diet of largemouth bass, *Micropterus salmoides*, in Lake Naivasha, Kenya. J Fish Biol. 1994;44: 607–619.

278. Pothoven SA, Vondracek B, Pereira DL. Effects of vegetation removal on bluegill and largemouth bass in two Minnesota lakes. North Am J Fish Manag. 1999;19: 748–757.

279. Middaugh CR, Foley CJ, Höök TO. Local and lake-scale habitat effects on abundance, lengths, and diets of age-0 largemouth bass and bluegill in Indiana temperate lakes. Trans Am Fish Soc. 2013;142: 1576–1589.

280. Swenson WA. Demographic changes in a largemouth bass population following closure of the fishery. American Fisheries Society Symposium. 2002. pp. 627–638.

281. Christensen DR, Moore BC. Differential prey selectivity of largemouth bass functional feeding groups in Twin Lakes, Washington. Lake Reserv Manag. 2007;23: 39–48.

282. Pine III WE, Ludsin SA, DeVries DR. First-summer survival of largemouth bass cohorts: Is early spawning really best? Trans Am Fish Soc. 2000;129: 504–513. doi:10.1577/1548-8659(2000)129<0504

283. Post DM, Kitchell JF, Hodgson JR. Interactions among adult demography, spawning date, growth rate, predation, overwinter mortality, and the recruitment of largemouth bass in a northern lake. Can J Fish Aquat Sci. 1998;55: 2588–2600.

284. Post DM. Individual variation in the timing of ontogenetic niche shifts in largemouth bass. Ecology. 2003;84: 1298–1310.

285. Johnson JM, Post DM. Morphological constraints on intracohort cannibalism in age-0 largemouth bass. Trans Am Fish Soc. 1996;125: 809–812.

286. Garvey JE, Wright RA, Stein RA. Overwinter growth and survival of age-0 largemouth bass (*Micropterus salmoides*): revisiting the role of body size. Can J Fish Aquat Sci. 1998;55: 2414–2424.

287. Gomiero LM, Villares-Jr GA, Naous F. Seasonal and ontogenetic variations in the diet of *Cichla kelberi* Kullander and Ferreira, 2006 introduced in an artificial lake in southeastern Brazil. Brazilian J Biol. 2010;8: 819–824. doi:10.1590/S1519-69842010000500017

288. Fugi R, Luz-Agostinho KDG, Agostinho AA. Trophic interaction between an introduced (peacock bass) and a native (dogfish) piscivorous fish in a Neotropical

impounded river. Hydrobiologia. 2008;607: 143–150. doi:10.1007/s10750-008-9384-2

289. Gomiero LM, Braga FM de S. Cannibalism as the main feeding behaviour of tucunares introduced in southeast Brazil. Brazilian J Biol. 2004;64: 625–632. doi:10.1590/S1519-69842004000400009

290. Santos LN dos, Gonzalez AF, Araújo FG. Diet of *Cichla monoculus* (Bloch & Schneider)(Osteichthyes, Cichlidae) in Lajes' Reservoir, Rio de Janeiro, Brazil. Rev Bras Zool. 2001;18: 191–204.

291. Novaes JLC, Caramaschi ÉP, Winemiller KO. Feeding of *Cichla monoculus* Spix, 1829 (Teleostei: Cichlidae) during and after reservoir formation in the Tocantins River, Central Brazil. Acta Limnol Bras. 2004;16: 41–49.

292. dos Santos AFGN, Santos LN dos, Andrade CC de, Araújo FG. Alimentação de duas espécies de peixes carnívoros no Reservatório de Lajes, RJ. Rev Univ Rural Sér Ci Vida Seropédica. 2004;24: 161–168.

293. Arcifa MS, Meschiatti AJ. Distribution and feeding ecology of fishes in a Brazilian reservoir: Lake Monte Alegre. Interciência-Caracas. 1993;18: 302.

294. Zaret TM. Inhibition of cannibalism in *Cichla ocellaris* and hypothesis of predator mimicry among South American fishes. Evolution (N Y). 1977;31: 421–437. doi:10.2307/2407762

295. Martins ML, Pereira-Jr J, De Chambrier A, Yamashita MM. Proteocephalid cestode infection in alien fish, *Cichla piquiti* Kullander and Ferreira, 2006 (Osteichthyes: Cichlidae), from Volta Grande reservoir, Minas Gerais, Brazil. Brazilian J Biol. 2009;69: 189–195.

296. Ward JA, Samarakoon JI. Reproductive tactics of the Asian cichlids of the genus *Etroplus* in Sri Lanka. Environ Biol Fishes. 1981;6: 95–103.

297. Sato T. Active accumulation of spawning substrate: a determinant of extreme polygyny in a shell-brooding cichlid fish. Anim Behav. 1994;48: 669–678.

298. Maan ME, Taborsky M. Sexual conflict over breeding substrate causes female expulsion and offspring loss in a cichlid fish. Behav Ecol. 2008;19: 302–308.

299. Schürch R, Heg D. Variation in helper type affects group stability and reproductive decisions in a cooperative breeder. Ethology. 2010;116: 257–269.

300. De Moor FC, Wilkinson RC, Herbst HM. Food and feeding habits of *Oreochromis mossambicus* (Peters) in hypertrophic Hartbeespoort Dam, South Africa. South African J Zool. 1986;21: 170–176.

301. Torres-Rojas YE, Hernandez Herrera A, Ortega-García S, Soto-Jiménez MF. Feeding habits variability and trophic position of dolphinfish in waters south of the Baja California Peninsula, Mexico. Trans Am Fish Soc. 2014;143: 528–542.

302. Moteki M, Arai M, Tsuchiya K, Okamoto H. Composition of piscine prey in the diet of large pelagic fish in the eastern tropical Pacific Ocean. Fish Sci. 2001;67: 1063–1074. doi:10.1046/j.1444-2906.2001.00362.x

303. Winemiller KO, Ponwith BJ. Comparative ecology of eleotrid fishes in Central American coastal streams. Environ Biol Fishes. 1998;53: 373–384.

304. Stott MK, Poulin R. Parasites and parental care in male upland bullies (Eleotridae). J Fish Biol. 1996;48: 283–291.

305. Bedarf AT, McKaye KR, Van Den Berghe EP, Perez LJ, Secor DH. Initial six-year expansion of an introduced piscivorous fish in a tropical Central American lake. Biol Invasions. 2001;3: 391–404.

306. Hishida Y. Egg consumption by the female in the paternal brooding goby *Bathygobius fuscus*. Fish Sci. 2002;68: 449–451. doi:10.1046/j.1444-2906.2002.00445.x

307. Shrivastava NP, Desai VR. A case of cannibalism observed in *Glossogobius giuris* (Hamilton) from Rihand Reservoir (Uttar Pradesh). Inl Fish Soc India. 1979;11.

308. Islam MN. Eco-biology of freshwater Gobi, *Glossogobius giuris* (Hamilton) of the river Padma in relation to its fishery: a review. J Biol Sci. 2004;4: 780–793.

309. Hernaman V, Probert PK, Robbins WD. Trophic ecology of coral reef gobies: interspecific, ontogenetic, and seasonal comparison of diet and feeding intensity. Mar Biol. 2009;156: 317–330. doi:10.1007/s00227-008-1085-x

310. Mazzoldi C, Poltronieri C, Rasotto MB. Egg size variability and mating system in the marbled goby *Pomatoschistus marmoratus* (Pisces: Gobiidae). Mar Ecol Prog Ser. 2002;233: 231–239.

311. Freitas V, Lika K, Witte JI, van der Veer HW. Food conditions of the sand goby *Pomatoschistus minutus* in shallow waters: An analysis in the context of Dynamic Energy Budget theory. J Sea Res. 2011;66: 440–446.

312. Pasquaud S, Girardin M, Elie P. Diet of gobies of the genus Pomatoschistus (*P. microps* and *P. minutus*), in the Gironde estuary (France). Cybium. 2004;28: 99–106.

313. Brandner J, Auerswald K, Cerwenka AF, Schliewen UK, Geist J. Comparative feeding ecology of invasive Ponto-Caspian gobies. Hydrobiologia. 2013;703: 113–131. doi:10.1007/s10750-012-1349-9

314. Adámek Z, Jurajda P, Prášek V, Sukop I. Seasonal diet pattern of non-native tubenose goby (*Proterorhinus semilunaris*) in a lowland reservoir (Mušov, Czech Republic). Knowl Manag Aquat Ecosyst. 2010;397: 12.

315. Takahashi D, Yanagisawa Y. Breeding ecology of an amphidromous goby of the genus *Rhinogobius*. Ichthyol Res. 1999;46: 185–191.

316. Ito S, Yanagisawa Y. Mate choice and cannibalism in a natural population of a stream goby, *Rhinogobius* sp. Ichthyol Res. 2000;47: 51–58. doi:10.1007/bf02674313

317. Taborsky M, Hudde B, Wirtz P. Reproductive behaviour and ecology of *Symphodus* (Crenilabrus) *ocellatus*, a European wrasse with four types of male behaviour. Behaviour. 1987;102: 82–117.

318. Nemtzov SC, Clark E. Intraspecific egg predation by male razorfishes (Labridae) during broadcast spawning: filial cannibalism or intra-pair parasitism? Bull Mar Sci. 1994;55: 133–141.

319. Russell DJ, Thuesen PA, Thomson FE, Power TN. Is stocking barramundi (*Lates calcarifer*) in north-eastern Queensland a threat to aquatic biodiversity? Mar Freshw Res. 2013;64: 992–1002.

320. Dadebo E, Mengistou S, Gebre-Mariam Z. Feeding habits of the Nile perch, *Lates niloticus* (L.)(Pisces: Centropomidae) in Lake Chamo, Ethiopia. SINET Ethipian J Sci. 2005;28: 61–68.

321. Katunzi EFB, Van Densen WLT, Wanink JH, Witte F. Spatial and seasonal patterns in the feeding habits of juvenile *Lates niloticus* (L.), in the Mwanza Gulf of Lake Victoria. Hydrobiologia. 2006;568: 121–133.

322. Kishe-Machumu MA, Witte F, Wanink JH, Katunzi EF. The diet of Nile perch, *Lates niloticus* (L.) after resurgence of haplochromine cichlids in the Mwanza Gulf of Lake Victoria. Hydrobiologia. 2012;682: 111–119.

323. McCawley JR, Cowan JH. Seasonal and size specific diet and prey demand of red snapper on Alabama artificial reefs. American Fisheries Society Symposium. 2007. p. 77.

324. Kelley D. Abundance, growth and first-winter survival of young bass in nurseries of south-west England. J Mar Biol Assoc United Kingdom. 2002;82: 307–319.

325. Moring JR, Mink LH. Anadromous alewives, *Alosa pseudoharengus*, as prey for white perch, *Morone americana*. Hydrobiologia. 2002;479: 125–130.

326. Carreon-Martinez L, Johnson TB, Ludsin SA, Heath DD. Utilization of stomach content DNA to determine diet diversity in piscivorous fishes. J Fish Biol. 2011;78: 1170–1182.

327. Gardinier MN, Hoff TB. Diet of striped bass in the Hudson River Estuary *Morone saxatilis*, New York. New York Fish Game J. 1982;26: 1486–1493.

328. Manojkumar PP, Pavithran PP, Ramachandran NP. Food and feeding habits of *Nemipterus japonicus* (Bloch) from Malabar coast, Kerala. Indian J Fish. 2015;62: 64–69.

329. Murilo C, Oyarzun C, Fernandez I. Latitudinal and temporal variation in the diet of *Dissostichus eleginoides* Smitt, 1898 (Perciformes: Nototheniidae) in deep environments of the south and center coast of Chile. Gayana. 2008;72: 94–101.

330. Pinkerton MH, Forman J, Bury SJ, Brown JA, Horn P, O’Driscoll RL. Diet and trophic niche of Antarctic silverfish *Pleuragramma antarcticum* in the Ross Sea,

Antarctica. J Fish Biol. 2013;82: 141–164.

331. Eastman JT. *Pleuragramma antarcticum* (Pisces, Nototheniidae) as food for other fishes in McMurdo Sound, Antarctica. Polar Biol. 1985;4: 155–160.

332. Koščo J, Manko P, Miklisová D, Košuthová L. Feeding ecology of invasive *Perccottus glenii* (Perciformes, Odontobutidae) in Slovakia. Czech J Anim Sci. 2008;53: 479–486.

333. Ferriz RA. Alimentacion de *Percichthys colhuapiensis* (Mac Donagh, 1955) y *P. trucha* (Girard, 1854) (Osteichthyes, Percichthyidae), en el Embaise Ramos Mexia, Provincia del Neuquen, Argentina. Iheringia. 1989;69: 109–116.

334. DeWoody JA, Fletcher DE, Wilkins SD, Avise JC. Genetic documentation of filial cannibalism in nature. Proc Natl Acad Sci. 2001;98: 5090–5092.

335. Liao H, Pierce CL, Larscheid JG. Consumption dynamics of the adult piscivorous fish community in Spirit Lake, Iowa. North Am J Fish Manag. 2004;24: 890–902.

336. Lewellen GR, White DA. The yellow perch fisheries of Deer Creek Reservoir, Utah, with notes on parasitism by Ligula intestinalis. Gt Basin Nat. 1971; 169–176.

337. Kövecses J, Sherwood GD, Rasmussen JB. Impacts of altered benthic invertebrate communities on the feeding ecology of yellow perch (*Perca flavescens*) in metal-contaminated lakes. Can J Fish Aquat Sci. 2005;62: 153–162.

338. Sanderson BL, Hrabik TR, Magnuson JJ, Post DM. Cyclic dynamics of a yellow perch (*Perca flavescens*) population in an oligotrophic lake: evidence for the role of intraspecific interactions. Can J Fish Aquat Sci. 1999;56: 1534–1542.

339. Hansson S, Post DM, Kitchell JF, McComish TS. Predation-competition interactions of yellow perch (*Perca flavescens*) and alewife (*Alosa pseudoharengus*) in southern Lake Michigan: A model analysis. Symp on the Role of Forage Fishes in Marine Ecosystems, Anchorage, Alaska, EEUU. 1996. pp. 13–16.

340. Woodland RJ, Secor DH. Benthic-pelagic coupling in a temperate inner continental shelf fish assemblage. Limnol Oceanogr. 2013;58: 966–976.

341. Zubenko EB. Seasonal feeding dynamics of the european perch, *Perca fluviatilis*, from the Kremenchug reservoir, Ukrainian - SSR, USSR. Vopr Ikhtiologii. 1979;19: 648–654.

342. Yerli S V., Alp A, Yegen V, Uysal R, Yagci MA, Balik I. Evaluation of the ecological and economical results of the introduced alien fish species in Lake Egirdir, Turkey. Turkish J Fish Aquat Sci. 2013;13: 795–809.

343. Magnhagen C, Hellström G, Borcherding J, Heynen M. Boldness in two perch populations–long-term differences and the effect of predation pressure. J Anim Ecol. 2012;81: 1311–1318.

344. Persson L, Byström P, Wahlström E. Cannibalism and competition in Eurasian perch: population dynamics of an ontogenetic omnivore. Ecology. 2000;81: 1058–1071. doi:10.1890/0012-9658(2000)081[1058:CACIEP]2.0.CO;2

345. Heynen M, Hellström G, Magnhagen C, Borcherding J. Does morphological variation between young-of-the-year perch from two Swedish lakes depend on genetic differences? Ecol Freshw Fish. 2010;19: 163–169.

346. Holmgren K, Appelberg M. Effects of environmental factors on size-related growth efficiency of perch, *Perca fluviatilis*. Ecol Freshw Fish. 2001;10: 247–256.

347. Lennmark I. From egg to predatory fish. Fauna och Flora Naturhistoriska Riksmuseet. 1985;80: 177–184.

348. Svanbäck R, Persson L. Individual diet specialization, niche width and population dynamics: implications for trophic polymorphisms. J Anim Ecol. 2004;73: 973–982.

349. Persson L, de Roos AM, Claessen D, Byström P, Lovgren J, Sjogren S, et al. Gigantic cannibals driving a whole-lake trophic cascade. Proc Natl Acad Sci U S A. 2003;100: 4035–4039. doi:10.1073/pnas.0636404100

350. Persson L, Andersson J, Wahlström E, Eklöv P. Size-specific interaction in lake

systems: predation gape limitation and prey growyh rate and mortality. Ecology. 1996;77: 900–911. doi:10.2307/2265510

351. Treasurer JW. Mortality and production of 0+ perch, *Perca fluviatilis* L., in two Scottish lakes. J Fish Biol. 1989;34: 913–928.

352. Treasurer JW. The population biology of perch, *Perca fluviatilis* L., in simple fish communities with no piscivore. Environ Biol Fishes. 1993;2: 16–22. doi:10.1111/j.1600-0633.1993.tb00012.x

353. K.S. D, Fedorova G V., Drozzhina KS. Daily nutritional rhythm of the pike perch *Stizostedion lucioperca* and the perch *Perca fluviatilis* Percidae in Lake Ladoga, Russian-SFSR USSR. Vopr Ikhtiologii. 1982; 224–232.

354. Barbara A. Fishes in the food of older perch (*Perca fluviatilis* L.) in lakes of the Wegorzewo district. Rocz Nauk Rol ser B Zootech. 1963;82: 273–294.

355. Mehner T, Schultz H, Bauer D, Herbst R, Voigt H, Benndorf J. Intraguild predation and cannibalism in age-0 perch (*Perca fluviatilis*) and age-0 zander (*Stizostedion lucioperca*): Interactions with zooplankton succession, prey fish availability and temperature. Ann Zool Fennici. 1996; 353–361.

356. Sabetian A, Trip EDL, Wheeler P, Sands L, Wakefield S, Visconti V, et al. Biological plasticity of non-native European perch (*Perca fluviatilis*) populations and the implications for management in northern New Zealand. New Zeal J Mar Freshw Res. 2015;49: 119–131.

357. Van Densen WLT, Ligtvoet W, Roozen RW. Intra-cohort variation in the individual size of juvenile pikeperch, *Stizostedion lucioperca*, and perch, *Perca fluviatilis*, in relation to the size spectrum of their food items. Ann Zool Fennici. 1996; 495–506.

358. Wang N, Eckmann R. Distribution of perch (*Perca fluviatilis* L.) during their first year of life in Lake Constance. Hydrobiologia. 1994;277: 135–143.

359. Beeck P, Tauber S, Kiel S, Borcherding J. 0+ perch predation on 0+ bream: a case study in a eutrophic gravel pit lake. Freshw Biol. 2002;47: 23459–2369.

360. Schleuter D, Eckmann R. Generalist versus specialist: the performances of perch

and ruffe in a lake of low productivity. Ecol Freshw Fish. 2008;17: 86–99.

361. Tolonen A, Lappalainen J, Pulliainen E. Seasonal growth and year class strength variations of perch near the northern limits of its distribution range. J Fish Biol. 2003;63: 176–186.

362. Goldspink CR, Goodwin D. A note on the age composition, growth rate and food of perch *Perca fluviatilis* (L.) in four eutrophic lakes, England. J Fish Biol. 1979;14: 489–505.

363. Smyly WJP. Observations on the food of the fry of perch (*Perca fluviatilis* Linn.) in Windermere. Proc Zool Soc London. 1952;122: 407–416.

364. Ohlberger J, Otero J, Edeline E, Winfield IJ, Stenseth NC, Vøllestad LA. Biotic and abiotic effects on cohort size distributions in fish. Oikos. 2013;122: 835–844.

365. Ohlberger J, Thackeray SJ, Winfield IJ, Aberly SC, Vøllestad LA. When phenology matters: age–size truncation alters population response to trophic mismatch. Proc R Soc London B. 2014;281: 2014–2038.

366. Holcik J. Changes in Fish Community of Klíčava Reservoir with Particular Reference to Eurasian Perch (*Perca fluviatilis*), 1957-72. J Fish Board Canada. 1977;34: 1734–1747.

367. Pivnička K. Long-termed study of fish populations in the Klicava Reservoir. Prirodoved Pr Ust Ceskoslov Akad Ved Brne. 1982; 1–46.

368. Bláha M, Šetlíkova I, Musil J, Policar T. No reason for keeping 0+ perch (*Perca fluviatilis* L.) with the prey fish. Aquac Int. 2013;21: 883–896.

369. Tarby MJ. Characteristics of yellow perch cannibalism in Oneida Lake and the relation to first year survival. Trans Am Fish Soc. 1974;103: 462–471.

370. Swenson WA, Smith-Jr LL. Influence of food competition, predation, and cannibalism on walleye (*Stizostedion vitreum vitreum*) and sauger (*S. canadense*) populations in Lake of the Woods, Minnesota. J Fish Board Canada. 1976;33: 1946–1954.

371. Özyurt CE, Mavruk S, Kiyağa VB. Effects of predator size and gonad maturation

on food preference and feeding intensity of *Sander lucioperca* (Linnaeus, 1758). Turkish J Fish Aquat Sci. 2012;12.

372. Balik I, Çubuk H, Karaşahin B, Özkök R, Uysal R, Ahmet ALP. Food and feeding habits of the pikeperch, *Sander lucioperca* (Linnaeus, 1758), population from Lake Eğirdir (Turkey). Turkish J Fish Aquat Sci. 2006;30: 19–26.

373. Campbell RNB. Food of an introduced population of pikeperch, *Stizostedion lucioperca* L., in lake Egirdir, Turkey. Aquac Res. 1992;23: 71–85.

374. Yilmaz M, Ablak Ö. The feeding behavior of pikeperch (*Sander lucioperca* (L., 1758)) living in Hirfanlı Dam Lake. Turkish J Vet Anim Sci. 2003;27: 1159–1165.

375. Balik İ. The feeding features of the pike-perch (*Stizostedion lucioperca*) population in Lake Beyşehir. Turkish J Zool. 1999;23: 189–194.

376. Pérez-Bote JL, Roso R. Diet of the introduced pikeperch *Sander lucioperca* (L.)(Osteichthyes, Percidae) in a recent colonised reservoir in south-western Iberian Peninsula. Ital J Zool. 2012;79: 617–626.

377. Frankiewicz P, Dabrowski K, Martyniak A, Zalewski M. Cannibalism as a regulatory force of pikeperch, Stizostedion lucioperca (L.), population dynamics in the lowland Sulejow reservoir (Central Poland). Shallow Lakes' 98a. Springer Netherlands; 1999. pp. 47–55.

378. Specziár A. First year ontogenetic diet patterns in two coexisting *Sander* species, *S. lucioperca* and *S. volgensis* in Lake Balaton. Hydrobiologia. 2005;549: 115–130.

379. Schulze T, Baade U, Dörner H, Eckmann R, Haertel-Borer SS, Hölker F, et al. Response of the residential piscivorous fish community to introduction of a new predator type in a mesotrophic lake. Can J Fish Aquat Sci. 2006;63: 2202–2212.

380. Dörner H, Hülsmann S, Hölker F, Skov C, Wagner A. Size-dependent predator-prey relationships between pikeperch and their prey fish. Ecol Freshw Fish. 2007;16: 307–314. doi:10.1111/j.1600-0633.2006.00223.x

381. Argillier C, Barral M, Irz P. Growth and diet of the pikeperch *Sander lucioperca*

(L.) in two French reservoirs. Arch Rybactwa Pol. 2003;11: 99–114.

382. Lappalainen J, Olin M, Vinni M. Pikeperch cannibalism: effects of abundance, size and condition. Ann Zool Fennici. 2006; 35–44.

383. Lappalainen J, Milardi M, Nyberg K, Venäläinen A. Effects of water temperature on year-class strengths and growth patterns of pikeperch (*Sander lucioperca* (L.)) in the brackish Baltic Sea. Aquat Ecol. 2009;43: 181–191.

384. Vinni M, Lappalainen J, Malinen T, Lehtonen H. Stunted growth of pikeperch *Sander lucioperca* in Lake Sahajärvi, Finland. J Fish Biol. 2009;74: 967–972.

385. Colby PJ, Lehtonen H. Suggested causes for the collapse of zander, *Stizostedion lucioperca*(L.), populations in northern and central Finland through comparisons with North American walleye, *Stizostedion vitreum* (Mitchill). Aqua Fenn. 1994;24: 9–20.

386. Kangur P, Kangur A, Kangur K. Dietary importance of various prey fishes for pikeperch *Sander lucioperca* (L.) in large shallow lake Võrtsjärv (Estonia). Proc Est Acad Sci Biol Ecol. 2007;56: 154–167.

387. Kocovsky PM, Carline RF. Dynamics of the unexploited walleye population of Pymatuning Sanctuary, Pennsylvania, 1997–1998. North Am J Fish Manag. 2001;21: 178–187.

388. Kempinger JJ, Carline RF. Dynamics of the walleye (*Stizostedion vitreum vitreum*) population in Escanaba Lake, Wisconsin, 1955-72. J Fish Board Canada. 1977;34: 1800–1811.

389. Hansen MJ, Bozek MA, Newby JR, Newman SP, Staggs MD. Factors affecting recruitment of walleyes in Escanaba Lake, Wisconsin, 1958–1996. North Am J Fish Manag. 1998;18: 764–774.

390. Forney JL. Year-class formation in the walleye (*Stizostedion vitreum vitreum*) population of Oneida Lake, New York, 1966-73. J Fish Board Canada. 1976;33: 783–792.

391. McElman JF, Balon KE. Early ontogeny of walleye, *Stizostedion vitreum*, with steps of saltatory development. Environ Biol Fishes. 1979;4: 309–348.

392. Johnston TA, Mathias JA. Mortality of first-feeding postlarval walleye (*Stizostedion vitreum*) in culture ponds. Can J Fish Aquat Sci. 1993;50: 1835–1843.

393. Loadman NL, Moodie GEE, Mathias JA. Significance of cannibalism in larval walleye (*Stizostedion vitreum*). Can J Fish Aquat Sci. 1986;43: 613–618.

394. Chevalier JR. Cannibalism as a factor in first year survival of walleye in Oneida Lake. Trans Am Fish Soc. 1973;102: 739–744.

395. Specziár A, Biro PA. Population structure and feeding characteristics of Volga pikeperch, *Sander volgensis* (Pisces, Percidae), in Lake Balaton. Hydrobiologia. 2003;506: 503–510.

396. Manica A. Alternative strategies for a father with a small brood: mate, cannibalise or care. Behav Ecol Sociobiol. 2002;2002: 4.

397. Cheney KL. Non-kin egg cannibalism and group nest-raiding by Caribbean sergeant major damselfish (*Abudefduf saxatilis*). Coral Reefs. 2008;27: 115.

398. Manica A. The effect of brood size and age on partial filial cannibalism in the scissortail sergeant. J Fish Biol. 2003;63: 37–47.

399. Nakazono A, Hamada H, Sakurai M. Predation on eggs by conspecific males in two Japanese damselfishes, *Pomacentrus nagasakiensis* and *Chromis notatus* notatus, after removal of egg-guarding males. J Ethol. 1989;7: 97–104.

400. Sikkel PC. Filial cannibalism in a paternal-caring marine fish: the influence of egg developmental stage and position in the nest. Anim Behav. 1994;47: 1149–1158.

401. Emslie MJ, Jones GP. Patterns of embryo mortality in a demersally spawning coral reef fish and the role of predatory fishes. Environ Biol Fishes. 2001;60: 363–373.

402. Payne AG, Smith C, Campbell AC. Filial cannibalism improves survival and development of beaugregory damselfish embryos. Proc R Soc London B. 2002;269: 2095–2102.

403. Hoelzer GA. Filial cannibalism in a non-brood cycling marine fish. Environ Biol Fishes. 1988;21: 309–313. doi:10.1007/BF00000378

404. Petersen CW, Marchetti K. Filial cannibalism in the Cortez damselfish *Stegastes rectifraenum*. Evolution (N Y). 1989;43: 158–168. doi:10.2307/2409171

405. Hoelzer GA. The ecology and evolution of partial-clutch cannibalism by paternal Cortez damselfish. Oikos. 1992;65: 113–120.

406. Munch SB, Conover DO. Recruitment dynamics of bluefish (*Pomatomus saltatrix*) from Cape Hatteras to Cape Cod, 1973–1995. ICES J Mar Sci. 2000;57: 393–402.

407. Akadje C, Diaby M, Le Loc'h F, Konan JK, N'Douba V. Diet of the barracuda *Sphyraena guachancho* in Côte d'Ivoire (Equatorial Eastern Atlantic Ocean). Cybium. 2013;37: 285–293.

408. Wuenschel MJ. Habitat and diet overlap of 4 piscivorous fishes: variation on the inner continental shelf off New Jersey. Fish Bull. 2013;111: 352–369.

409. Acha EM, Bremec C, Lasta C. Cannibalism on planktonic eggs by a non-filter feeding fish, *Micropogonias furnieri* (Sciaenidae). Fish Res. 2002;56: 321–326. doi:10.1016/S0165-7836(01)00326-5

410. Stefani PM, Rocha O. Diet composition of *Plagioscion squamosissimus* (Heckel, 1840), a fish introduced into the Tietê River system. Brazilian. Brazilian J Biol. 2009;69: 805–812.

411. Hahn NS, Agostinho AA, Goitein R. Feeding ecology of curvina *Plagioscion squamosissimus* (Heckel, 1840)(Osteichthyes, Perciformes) in the Itaipu reservoir and Porto Rico floodplain. Acta Limnol Bras. 1997;9: 11–22.

412. Izuka K, Asano M, Naganuma A. Feeding habits of skipjack tuna (*Katsuwonus pelamis* Linnaeus) caught by pole and line and the state of young skipjack tuna distribution in the tropical seas of the western Pacific Ocean. Bull Tohoku Reg Fish Res Lab. 1989;

413. Nakamura EL. Food and feeding habits of skipjack tuna (*Katsuwonus pelamis*) from the Marquesas and Tuamotu Islands. Trans Am Fish Soc. 1965;94: 236–

242.

414. Glaser SM. Interdecadal variability in predator-prey interactions of juvenile North Pacific albacore in the California Current System. Mar Ecol Prog Ser. 2010;414: 209–221.

415. Young JW. Feeding ecology of larvae of southern bluefin, albacore and skipjack tunas (Pisces: Scombridae) in the eastern Indian Ocean. Mar Ecol Prog Ser. 1990;61: 17–29.

416. Reglero P, Urtizberea A, Torres AP, Alemany F, Fiksen Ø. Cannibalism among size classes of larvae may be a substantial mortality component in tuna. Mar Ecol Prog Ser. 2011;433: 205–219.

417. Fortier L, Villeneuve A. Cannibalism and predation on fish larvae by larvae of Atlantic mackerel, *Scomber scombrus*: trophodynamics and potential impact on recruitment. Fish Bull. 1996;94: 268–281.

418. Peterson WT, Ausubel SJ. Diets and selective feeding by larvae of Atlantic mackerel *Scomber scombrus* on zooplankton. Mar Ecol Prog Ser. 1984;17: 65–75.

419. Hillgruber N, Kloppmann M, Wahl E, Westernhagen H V. Feeding of larval blue whiting and Atlantic mackerel: a comparison of foraging strategies. J Fish Biol. 1997;51: 230–249.

420. Hillgruber N, Kloppmann M. Small-scale patterns in distribution and feeding of Atlantic mackerel (*Scomber scombrus* L.) larvae in the Celtic Sea with special regard to intra-cohort cannibalism. Helgol Mar Res. 2001;55: 135–149.

421. Paradis V, Sirois P, Castonguay M, Plourde S. Spatial variability in zooplankton and feeding of larval Atlantic mackerel (*Scomber scombrus*) in the southern Gulf of St. Lawrence. J Plankton Res. 2012;34: 1064–1077.

422. Condini M V., Hoeinghaus DJ, Garcia AM. Trophic ecology of dusky grouper *Epinephelus marginatus* (Actinopterygii, Epinephelidae) in littoral and neritic habitats of southern Brazil as elucidated by stomach contents and stable isotope analyses. Hydrobiologia. 2015;743: 109–125.

423. Letourneur Y, Chabanet P, Vigliola L, Harmelin-Vivien M. Mass settlement and post-settlement mortality of *Epinephelus merra* (Pisces: Serranidae) on Reunion coral reefs. J Mar Biol Assoc United Kingdom. 1998;78: 307–319.

424. Klimpel S, Rückert S, Piatkowski U, Palm HW, Hanel R. Diet and metazoan parasites of silver scabbard fish *Lepidopus caudatus* from the Great Meteor Seamount (North Atlantic). Mar Ecol Prog Ser. 2006;315: 249–257.

425. Liu Y, Cheng J, Chen Y. A spatial analysis of trophic composition: a case study of hairtail (*Trichiurus japonicus*) in the East China Sea. Hydrobiologia. 2009;632: 79–90.

426. De la Cruz-Torres J, Martínez-Pérez JA, Franco-López J, Ramírez-Villalobos AJ. Biological and Ecological Aspects of *Trichiurus lepturus* Linnaeus, 1758 (Perciformes: Trichiuridae) in Boca Del Rio, Veracruz, Mexico. Am J Agric Environ Sci. 2014;14: 1058–1066.

427. Martins AS, Haimovici M, Palacios R. Diet and feeding of the cutlassfish Trichiurus lepturus in the Subtropical Convergence Ecosystem of southern Brazil. J Mar Biol Assoc United Kingdom. 2005;85: 1223–1229. doi:10.1017/S002531540501235X

428. Bittar VT, Di Denedito APM. Diet and potential feeding overlap between *Trichiurus lepturus* (Osteichthyes: Perciformes) and *Pontoporia blainvillei* (Mammalia: Cetacea) in northern Rio de Janeiro, Brazil. Zoologia. 2009;26: 374–378.

429. Omori M, Seino Y. Feeding preference of the hairtail *Trichiurus lepturus* linnaeus in and neighbouring the waters where Sergia lucens swarms in Suruga bay Japan. Bull Japanese Soc Sci Fish. 1993;57.

430. Ghosh S, Rao M V., Rohit P, Rammohan K, Maheswarudu G. Reproductive biology, trophodynamics and stock structure of ribbonfish *Trichiurus lepturus* from northern Arabian Sea and northern Bay of Bengal. Indian J Geo-Marine Sci. 2014;43: 755–771.

431. Lin L, Zhang H, Li H, Cheng J. Study on seasonal variation of the feeding habits of hairtail (*Trichiurus japonicus*) in the East China Sea. Period Ocean Univ

China. 2006;36: 932–936.

432. Lin LS, Chen JH, Li HY. The fishery biology of *Trichiurus japonicus* and *Larimichthys polyactis* in the East China Sea region. Mar Fish. 2008;30: 126–134.

433. Yana Y, Chena J, Lua H, Houa G, Lai J. Feeding habits and ontogenetic diet shifts of hairtail, *Trichiurus margarites*, in the Beibu Gulf of the South China Sea. Acta Ecol Sin. 2012;32: 18–25.

434. Ohta T, Nakazono A. Mating habits, mating system and possible filial cannibalism in the triplefin, *Enneapterygius etheostomus*. Proceedings of the 6th International Coral Reef Symposium, Australia. 1988. pp. 797–801.

435. Hamada H, Nakazono A. Reproductive ecology of the triplefin, *Enneapterygius etheostomus*, with special reference to the occurrence of fish eggs in the digestive tract of the male. Sci Bull Fac Agric Univ. 1989;43.

436. Castillo-Rivera BM, Kobelkowsky A, Chavez AM. Feeding biology of the flatfish *Citharichthys spilopterus* (Bothidae) in a tropical estuary of Mexico. J Appl Ichthyol. 2000;16: 73–78.

437. Tanaka M, Goto T, Tomiyama M, Sudo H. Immigration, settlement and mortality of flounder (*Paralichthys olivaceus*) larvae and juveniles in a nursery ground, Shijiki Bay, Japan. Netherlands J Sea Res. 1989;24: 57–67.

438. Noichi T, Kusano M, Kanbara T, Senta T. Predation by fishes on larval and juvenile japanese flounder at Yanagihama Beach, Nagasaki, Japan. Nippon Suisan Gakkaishi. 1993;59: 1851–1855.

439. Furuta S. Seasonal changes in abundance, length distribution, feeding condition and predation vulnerability of juvenile Japanese flounder, *Paralichthys olivaceus*, and prey mysid density in the Tottori coastal area Japan. Bull Japanese Soc Sci Fish. 1999;65: 167–174.

440. Oshima M, Robert D, Kurita Y, Yoneda M, Tominaga O, Tomiyama T, et al. Do early growth dynamics explain recruitment success in Japanese flounder *Paralichthys olivaceus* off the Pacific coast of northern Japan? J Sea Res.

2010;64: 94–101.

441. Norbis W, Galli O. Feeding habits of the flounder *Paralichthys orbignyanus* (Valenciennes, 1842) in a shallow coastal lagoon of the southern Atlantic Ocean: Rocha, Uruguay. Ciencias Mar. 2004;30: 619–625.

442. Selleslagh J, Amara R. Are estuarine fish opportunistic feeders? The case of a low anthropized nursery ground (the Canche Estuary, France). Estuaries and Coasts. 2015;38: 252–267.

443. Román E, González C, Paz X. Condition and feeding of Greenland Halibut (*Reinhardtius hippoglossoides*) in the North Atlantic with emphasis on the Flemish Cap. J Northwest Atl Fish Sci. 2007;37: 165–179.

444. Rodriguez-Marin E, Punzón A, Paz J. Feeding patterns of Greenland halibut (*Reinhardtius hippoglossoides*) in Flemish Pass (northwest Atlantic). NAFO Sci Counc Stud. 1995;23: 43–54.

445. Solmundsson K. Trophic ecology of Greenland halibut (*Reinhardtius hippoglossoides*) on the Icelandic continental shelf and slope. Mar Biol Res. 2007;3: 231–242.

446. St.-Pierre G. Recent studies of Pacific halibut postlarvae in the Gulf of Alaska and eastern Bering Sea. Seattle, Wash Int Pacific Halibut Comm. 1989;

447. Hanson JM, Wilson T. Abundance, distribution, and diet of a small-bodied ecotype of Windowpane. Trans Am Fish Soc. 2014;143: 650–659.

448. Hoff MH. Biotic and abiotic factors related to lake herring recruitment in the Wisconsin waters of Lake Superior, 1984–1998. J Great Lakes Res. 2004;30: 423–433.

449. Skurdal J, Bleken E, Stenseth NC. Cannibalism in whitefish (*Coregonus lavaretus*). Oecologia. 1985;67: 566–571.

450. Straile D, Eckmann R, Jüngling T, Thomas G, Löffler H. Influence of climate variability on whitefish (*Coregonus lavaretus*) year-class strength in a deep, warm monomictic lake. Oecologia. 2007;151: 521–529.

451. Amundsen P-A, Krisroffersen R, Knudsen R, Klemetsen A. Long-term effects of a stock depletion programme: the rise and fall of a rehabilitated whitefish population. Adv Limnol. 2001;57: 577–588.

452. Propst DL, Stefferud JA. Population dynamics of Gila trout in the Gila River drainage of the south-western United States. J Fish Biol. 1997;51: 1137–1154.

453. Rinne JN. Spawning habitat and behavior of Gila trout, a rare salmonid of the southwestern United States. Trans Am Fish Soc. 1980;109: 83–91.

454. Krkošek M, Hilborn R, Peterman RM, Quinn TP. Cycles, stochasticity and density dependence in pink salmon population dynamics. Proc R Soc London B. 2011;278: 2060–2068.

455. Barber FG. Pink Salmon *Oncorhynchus gorbuscha* east. Can Tech Rep Fish Aquat Sci. 1981;986: 1–8.

456. Ricker WE. Regulation on the abundance of pink Salmon (Oncorhynchus gorbuscha). J Fish Res Board Canada. 1963;713.

457. Roseman EF, Schaeffer JS, Bright E, Fielder DG. Angler-caught piscivore diets reflect fish community changes in Lake Huron. Trans Am Fish Soc. 2014;143: 1419–1433.

458. Huryn AD. An appraisal of the Allen paradox in a New Zealand trout stream. Limnol Oceanogr. 1996;41: 243–252.

459. O’Brien TP, Taylor WW, Briggs AS, Roseman EF. Influence of water temperature on rainbow smelt spawning and early life history dynamics in St. Martin Bay, Lake Huron. J Great Lakes Res. 2012;38: 776–785.

460. Sepulveda AJ, Lowe WH, Marra PP. Using stable isotopes to test for trophic niche partitioning: a case study with stream salamanders and fish. Freshw Biol. 2012;57: 1399–1409.

461. Sagawa S, Yamashita S, Satou K, Nakamura F. Fall habitat use and foraging mode of immature Sakhalin taimen in the river tributaries in northern Hokkaido, Japan. Japanese J Ecol. 2003;53.

462. Symons PEK, Heland M. Stream habitats and behavioral interactions of underyearling and yearling Atlantic salmon (*Salmo salar*). J Fish Board Canada. 1978;35: 175–183.

463. Vik JO, Borgstrøm R, Skaala O. Cannibalism governing mortality of juvenile brown trout, *Salmo trutta*, in a regulated stream. Regul Rivers Res Manag. 2001;17: 583–594.

464. Jensen H, Kiljunen M, Amundsen P-A. Dietary ontogeny and niche shift to piscivory in lacustrine brown trout *Salmo trutta* revealed by stomach content and stable isotope analyses. J Fish Biol. 2012;80: 2448–2462.

465. Salavatian M, Gholiev Z, Aliev A, Abassi K. Feeding behavior of brown trout, *Salmo trutta fario*, during spawning season in four rivers of Lar National Park, Iran. Casp J Environ Sci. 2011;9: 223–233.

466. Grey J, Thackeray SJ, Jones RI, Shine A. Ferox Trout (*Salmo trutta*) as Russian dolls': complementary gut content and stable isotope analyses of the Loch Ness foodweb. Freshw Biol. 2002;47: 1235–1243. doi:10.1046/j.1365-2427.2002.00838.x

467. Tentelier C, Larrieu M, Aymes JC, Labonne J. Male antagonistic behaviour after spawning suggests paternal care in brown trout, *Salmo trutta*. Ecol Freshw Fish. 2011;20: 580–587.

468. Aymes JC, Larrieu M, Tentelier C, Labonne J. Occurrence and variation of egg cannibalism in brown trout *Salmo trutta*. Naturwissenschaften. 2010;97: 435–439.

469. Borgstrøm R, Brittaun JE, Hasle K, Skjølås S, Dokk JG. Reduced recruitment in brown trout *Salmo trutta*, the role of interactions with the minnow *Phoxinus phoxinus*. Nord J Freshw Res. 1996;72: 30–38.

470. Borgstrøm R, Museth J, Brittaun JE. The brown trout (*Salmo trutta*) in the lake, Øvre Heimdalsvatn: long-term changes in population dynamics due to exploitation and the invasive species, European minnow (*Phoxinus phoxinus*). The subalpine lake ecosystem, Øvre Heimdalsvatn, and its catchment: local and global changes over the last 50 years. Springer Netherlands; 2010. pp. 81–91.

471. Hammar J. Natural resilience in Arctic charr *Salvelinus alpinus*: life history, spatial and dietary alterations along gradients of interspecific interactions. J Fish Biol. 2014;85: 81–118.

472. Borgstrøm R, Isdahl T, Svenning M-A. Population structure, biomass, and diet of landlocked Arctic charr (*Salvelinus alpinus*) in a small, shallow High Arctic lake. Polar Biol. 2015;38: 309–317. doi:10.1007/s00300-014-1587-6

473. Byström P, Andersson J, Persson L, de Roos AM. Size-dependent resource limitation and foraging-predation risk trade-offs: growth and habitat use in young arctic char. Oikos. 2004;104: 109–121.

474. Damsgård B, Ugedal O. The influence of predation risk on habitat selection and food intake by Arctic charr, *Salvelinus alpinus* (L.). Ecol Freshw Fish. 1997;6: 95–101.

475. Amundsen P-A. Piscivory and cannibalism in Arctic charr. J Fish Biol. 1994;45: 181–189. doi:10.1006/jfbi.1994.1222

476. Finstad AG, Jansen PA, Langeland L. Production and predation rates in a cannibalistic arctic char (*Salvelinus alpinus* L.) population. Ecol Freshw Fish. 2001;10: 220–226.

477. Berg OK, Finstad AG, Olsen PH, Arnekleiv JV, Nilssen K. Dwarfs and cannibals in the Arctic: production of Arctic char (*Salvelinus alpinus* (L.)) at two trophic levels. Hydrobiologia. 2010;652: 337–347. doi:10.1007/s10750-010-0366-9

478. Riget FF, Nygaard KH, Christensen B. Population structure, ecological segregation, and reproduction in a population of Arctic char (*Salvelinus alpinus*) from Lake Tasersuaq, Greenland. Can J Fish Aquat Sci. 1986;43: 985–992.

479. Power ME, Power G, Reist JD, Bajno R. Ecological and genetic differentiation among the Arctic charr of Lake Aigneau, Northern Québec. Ecol Freshw Fish. 2009;18: 445–460.

480. Gantner N, Power ME, Iqaluk D, Meili M, Borg H, Sundbom M, et al. Mercury concentrations in landlocked Arctic char (*Salvelinus alpinus*) from the Canadian Arctic. Part I: insights from trophic relationships in 18 lakes. Environ Toxicol

Chem. 2010;29: 621–632.

481. Gantner N, Veillette J, Michaud WK, Bajno R, Muir D, Vincent WF, et al. Physical and biological factors affecting mercury and perfluorinated contaminants in Arctic char (*Salvelinus alpinus*) of Pingualuit Crater Lake (Nunavik, Canada). Arctic. 2012; 195–206.

482. Hobson KA, Welch HE. Cannibalism and trophic structure in a high Arctic lake: insights from stable-isotope analysis. Can J Fish Aquat Sci. 1995;52: 1195–1201.

483. Sánchez-Hernández J, Amundsen P-A. Trophic ecology of brown trout (*Salmo trutta* L.) in subarctic lakes. Ecol Freshw Fish. 2015;24: 148–161.

484. Beauchamp DA, Van Tassel JJ. Modeling seasonal trophic interactions of adfluvial bull trout in Lake Billy Chinook, Oregon. Trans Am Fish Soc. 2001;130: 204–216.

485. Gutowsky LF, Harrison PM, Martins EG, Leake A, Patterson DA, Power ME, et al. Diel vertical migration hypotheses explain size-dependent behaviour in a freshwater piscivore. Anim Behav. 2013;86: 365–373.

486. Pinto MC, Post JR, Paul AJ, Johnston FD, Mushens CJ, Stelfox JD. Lateral and longitudinal displacement of stream-rearing juvenile Bull Trout in response to upstream migration of spawning adults. Trans Am Fish Soc. 2013;142: 1590–1601.

487. Blanchfield PJ, Ridgway MS. The cost of peripheral males in a brook trout mating system. Anim Behav. 1999;57: 537–544.

488. Maekawa K, Hino T. Spawning tactics of female Miyabe charr (*Salvelinus malma miyabei*) against egg cannibalism. Can J Zool. 1990;68: 889–894.

489. Maekawa K. Streaking behaviour of mature male parrs of the Miyabe charr, Salvelinus malma miyabei, during spawning. Gyoruigaku Zasshi. 1983;30.

490. Martin N V. A study of the lake trout, *Salvelinus namaycush*, in two Algonquin Park, Ontario, lakes. Trans Am Fish Soc. 1952;81: 111–137.

491. Eshenroder RL, Crossman EJ, Meffe GK, Olver CH, Pister EP. Lake trout

rehabilitation in the Great Lakes: an evolutionary, ecological, and ethical perspective. J Great Lakes Res. 1995;21.

492. Madenjian CP, Desorcie TJ, Stedman RM. Ontogenic and spatial patterns in diet and growth of lake trout in Lake Michigan. Trans Am Fish Soc. 1998;127: 236–252.

493. Elrod JH. Survival of hatchery-reared lake trout stocked near shore and off shore in Lake Ontario. North Am J Fish Manag. 1997;17: 779–783.

494. Gamble AE, Hrabik TR, Yule DL, Stockwell JD. Trophic connections in Lake Superior Part II: the nearshore fish community. J Great Lakes Res. 2011;37: 550–560.

495. Mitrofanov VP, Petr T. Fish and fisheries in the Altai, Northern Tien Shan and Lake Balkhash (Kazakhstan). Fish Fish High Altitudes Asia, FAO Fish Tech Pap. 1999;385: 149–167.

496. Koczaja C, McCall L, Fitch E, Glorioso B, Hanna C, Kyzar J, et al. Size-specific habitat segregation and intraspecific interactions in banded sculpin (*Cottus carolinae*). Southeast Nat. 2005;4: 107–218.

497. Marconato A, Bisazza A. Mate choice, egg cannibalism and reproductive success in the river bullhead, *Cottus gobio* L. J Fish Biol. 1988;33: 905–916.

498. Marconato A, Bisazza A, Fabris M. The cost of parental care and egg cannibalism in the river bullhead, *Cottus gobio* L.(Pisces, Cottidae). Behav Ecol Sociobiol. 1993;32: 229–237.

499. Goto A. Male mating success and female mate choice in the river sculpin, *Cottus nozawae* (Cottidae). Environ Biol Fishes. 1993;37: 347–353.

500. Johnston CE. Allopaternal care in the Pygmy Sculpin (*Cottus pygmaeus*). Copeia. 2000; 262–264.

501. Kobler A, Humblet Y, Geudens K, Eens M. Period-dependent sex-biased movement in a polygamous stream fish (*Cottus perifretum* Freyhof, Kottelat & Nolte, 2005—Actinopterygii, Cottidae) with male parental care. Hydrobiologia. 2012;693: 195–204.

502. Yamada K, Hori M, Tanaka Y, Hasegawa N, Nakaoka M. Contribution of different functional groups to the diet of major predatory fishes at a seagrass meadow in northeastern Japan. Estuar Coast Shelf Sci. 2010;86: 71–82.

503. Hauksson E. Studies on the diet of short spined sea scorpion *Myoxocephalus scorpius* in Icelandic waters. Hafranns Fjolrit. 2005;115: 17–20.

504. Mychek-Londer JG, Bunnell DB, Stott W, Diana JS, French III JR, Chriscinske MA. Using diets to reveal overlap and egg predation among benthivorous fishes in Lake Michigan. Trans Am Fish Soc. 2013;142: 492–504.

505. Munehara H, Miura T. Non-intentional filial egg cannibalism by the guarding male of *Hexagrammos otakii* (Pisces: Hexagramidae). J Ethol. 1995;13: 191–193.

506. King JR, Withler RE. Male nest site fidelity and female serial polyandry in lingcod (*Ophiodon elongatus*, Hexagrammidae). Mol Ecol. 2005;14: 653–660.

507. DeMartini EE. Paternal defence, cannibalism and polygamy: factors influencing the reproductive success of painted greenling (Pisces, Hexagrammidae). Anim Behav. 1987;35: 1145–1158.

508. Rand KM, Lowe SA. Defining essential fish habitat for Atka mackerel with respect to feeding within and adjacent to Aleutian Islands trawl exclusion zones. Mar Coast Fish. 2011;3: 21–31.

509. Zolotov OG. Some biological features of the reproduction of the Atka mackerel *Pleurogrammus monopterygius* in coastal waters of Kamchatka. Vopr Ikhtiologii. 1992;32: 110–119.

510. Lauth RR, Guthridge JL, Nichol D, McEntire SW, Hillgruber N. Timing and duration of mating and brooding periods of Atka mackerel (*Pleurogrammus monopterygius*) in the North Pacific Ocean. Fish Bull. 2007;105: 560–570.

511. Valdez-Moreno M, Quintal-Lizama C, Gómez-Lozano R, García-Rivas MDC. Monitoring an alien invasion: DNA barcoding and the identification of lionfish and their prey on coral reefs of the Mexican Caribbean. PLoS One. 2012;7: e36636.

512. Carmo V, Sutton T, Menezes G, Falkenhaug T, Bergstad OA. Feeding ecology of the Stomiiformes (Pisces) of the northern Mid-Atlantic Ridge. 1. The Sternoptychidae and Phosichthyidae. Prog Oceanogr. 2015;130: 172–187.

513. Sandhu AA, Lone KP. Food and feeding habits of some catfishes of Pakistan. Pak J Zool. 2003;35: 353–356.

514. Whitfield AK, Blaber SJM. Food and feeding ecology of piscivorous fishes at Lake St Lucia, Zululand. J Fish Biol. 1978;13: 675–691.

515. Mosepele K, Mosepele B, Wolski P, Kolding J. Dynamics of the feeding ecology of selected fish species from the Okavango River delta, Botswana. Acta Ichthyol Piscat. 2012;42: 271–289.

516. Baumann JR, Kwak TJ. Trophic relations of introduced flathead catfish in an Atlantic river. Trans Am Fish Soc. 2011;140: 1120–1134.

517. Copp GH, Robert Britton J, Cucherousset J, García-Berthou E, Kirk, R., Peeler E, Stakėnas S. Voracious invader or benign feline? A review of the environmental biology of European catfish *Silurus glanis* in its native and introduced ranges. Fish Fish. 2009;10: 252–282.

518. Bora ND, Gül A. Feeding biology of Silurus glanis (L., 1758) Living in Hirfanl› Dam Lake. Turkish J Vet Anim Sci. 2004;28: 471–479.

519. Kitsos MS, Tzomos TH, Anagnostopoulou L, Koukouras A. Diet composition of the seahorses, *Hippocampus guttulatus* Cuvier, 1829 and *Hippocampus hippocampus* (L., 1758)(Teleostei, Syngnathidae) in the Aegean Sea. J Fish Biol. 2008;72: 1259–1267.

520. Storero LP, González RA. Feeding habits of the seahorse *Hippocampus patagonicus* in San Antonio Bay (Patagonia, Argentina). J Mar Biol Assoc United Kingdom. 2008;88: 1503–1508.

521. Teixeira RL, Musick JA. Trophic ecology of two congeneric pipefishes (Syngnathidae) of the lower York River, Virginia. Environ Biol Fishes. 1995;43: 295–309.

522. Malavasi S, Riccato F, Georgalas V, Franzoi P, Torricelli P. Occurrence and

intensity of intercohort cannibalism of post-hatching stages in the broad-nosed pipefish, *Syngnathus typhle*. J Appl Ichthyol. 2009;25: 622.

523. Nakazono A, Kawase H. Spawning and biparental egg-care in a temperate filefish, *Paramonacanthus japonicus* (Monacanthidae). Environ Biol Fishes. 2003;37: 245–256.
