# Supplementary material for: Is There a Relationship between Fish Cannibalism and Latitude or Species Richness?
Source: PLoS One. 2017 Jan 25;12(1):e0169813. doi: 10.1371/journal.pone.0169813 (PMC5266261; doi:10.1371/journal.pone.0169813)
Supplement: S4 Table — Number of records identified, included and excluded, and the reasons for exclusions for the systematic review and meta-analysis. (DOC) [file pone.0169813.s005.doc]

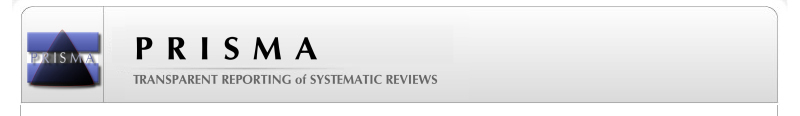
**PRISMA 2009 Flow Diagram**

**Screening**

**Included**

**Eligibility**

**Identification**

Records identified through database searching
(n = 2123 )

Additional records identified through other sources
(n = 0 )

Records after duplicates removed
(n = 2078 )

Records screened
(n = 2078 )

Records excluded
(n = 0 )

Full-text articles assessed for eligibility
(n = 2044 )

Full-text articles excluded, with reasons
(n = 777 )

Studies included in qualitative synthesis
(n = 1267 )

Studies included in quantitative synthesis (meta-analysis)
(n = 1267 )
